# In Vitro and In Vivo Characterization Methods for Evaluation of Modern Wound Dressings

**DOI:** 10.3390/pharmaceutics15010042

**Published:** 2022-12-22

**Authors:** Naveed Ahmad

**Affiliations:** Department of Pharmaceutics, College of Pharmacy, Jouf University, Sakaka 72388, Aljouf, Saudi Arabia; nakahmad@ju.edu.sa or naveedpharmacist@yahoo.com

**Keywords:** chronic wounds, modern wound dressings, wound healing, wound management, in vitro healing assay, in vivo wound healing models

## Abstract

Chronic wound management represents a major challenge in the healthcare sector owing to its delayed wound-healing process progression and huge financial burden. In this regard, wound dressings provide an appropriate platform for facilitating wound healing for several decades. However, adherent traditional wound dressings do not provide effective wound healing for highly exudating chronic wounds and need the development of newer and innovative wound dressings to facilitate accelerated wound healing. In addition, these dressings need frequent changing, resulting in more pain and discomfort. In order to overcome these issues, a wide range of affordable and innovative modern wound dressings have been developed and explored recently to accelerate and improve the wound healing process. However, a comprehensive understanding of various in vitro and in vivo characterization methods being utilized for the evaluation of different modern wound dressings is lacking. In this context, an overview of modern dressings and their complete in vitro and in vivo characterization methods for wound healing assessment is provided in this review. Herein, various emerging modern wound dressings with advantages and challenges have also been reviewed. Furthermore, different in vitro wound healing assays and in vivo wound models being utilized for the evaluation of wound healing progression and wound healing rate using wound dressings are discussed in detail. Finally, a summary of modern wound dressings with challenges and the future outlook is highlighted.

## 1. Introduction

The skin represents the largest organ and constitutes 16% of total body weight as it covers the entire human body [1,2]. The major functions of the skin include a protective barrier against dehydration and the external environment (pathogens, physical agents, and chemical agents) [3]. In addition, skin functions also include temperature regulation, water loss prevention, vitamin D3 synthesis, and sensory functions [4]. Therefore, cutaneous damage (wounds) in the form of cuts, burns, skin diseases, surgical incisions, metabolic disorders (diabetes), and others hugely affect the skin structure and function and pose severe human health risks. As soon as the injury occurs, restoration of skin structure and functions are mediated by the wound healing process through distinct coordinated phases, viz., hemostasis, inflammation, proliferation, and remodeling through interactions of various cells, growth factors, and cytokines [5]. The wound healing process starts immediately, in the case of acute wounds, after injury to ensure body homeostasis and avoid bacterial contamination. The distinction of wounds into acute and chronic wounds relies on the success of wound healing. Unlike acute wounds, chronic (non-healing) wounds do not follow the traditional wound-healing process in an orderly and timely manner and stall at the inflammation phase paving the way for infections. Chronic wounds represent wounds with pre-existing pathological conditions (autoimmune diseases, diabetes, venous stasis) which take more than three months to heal, impair an individual’s quality of life, and pose huge financial and social challenges with increased motility and morbidity [6,7]. As per recent statistics, approximately 2.5% of the US population’s quality of life is negatively affected by chronic wounds owing to clinical complications, social challenges, and economic burden. It is expected that ten out of a thousand people will experience chronic wounds in their lifetime [8,9]. The prevalence of chronic wounds (silent epidemic) is further aggravated by growing numbers of the aging population and chronic diseases such as obesity, diabetes, chronic kidney disease, cancer, etc. [10,11]. As per earlier reports over $25 billion is being spent annually in the USA alone for chronic wound management [9,12,13]. Moreover, the increasing incidences of chronic wound conditions with aging and metabolic diseases necessitate the need for the development of effective and innovative strategies of robust nature for the optimization of healthcare management. The developed strategies and approaches would intend to diminish the health and economic burdens by addressing the multicellular intricate mechanisms for quality wound healing.

A variety of strategies and approaches have been extensively employed for improved skin wound healing, however, owing to the difficulty in assessment and wound care management, chronic wound management remains as the unmet therapeutic area [14,15]. Among the effective and innovative strategies, novel wound dressings have a large global impact as applicable wound care products for promotion and acceleration of the complex wound healing process. Over the years, numerous wound dressings in the form of gauze, bandages, foams, and hydrogels have been developed and translated to clinics for wound closure and skin tissue regeneration in various kinds of wounds [16,17,18]. The dressings should fulfill certain criteria: (1) Ability to provide a moist environment; (2) Exhibit mechanical stability against stress and pressure; (3) Facilitate cell migration and epithelialization; (4) Ability to show low adherence to the wounds; (5) Capability to maintain elastic texture and flexibility to adapt to the specific wound conditions; (6) Most importantly, act as a protective barrier against external threats [19,20]. Though conventional wound dressings are highly absorbent in nature and effective for dry to mild exudating wounds, they still suffer from various limitations and challenges, especially in the case of chronic wounds, wherein abnormal changes occur during the wound healing process. The lack of modulation of properties according to the changing wound conditions poses a major challenge in the usage of conventional wound dressings. In addition, traditional wound dressings do not provide enough wound drainage, require frequent changes associated with pain, and lack desired mechanical properties, adhesion properties, stimuli-responsiveness properties, and inherent bioactivity. Furthermore, additional challenges are presented by the large volume of wound exudates associated with chronic wounds present additional challenges [21]. Therefore, there is an urgent need to normalize the healing process through utilization of wound dressings with the ability to address the abnormal changes happening during chronic wound healing for effective and timely wound treatment. Modern wound dressings are being explored and implemented in recent years to overcome the limitations of traditional wound dressings by facilitating an accelerated wound healing process through enhanced cell migration, granulation tissue formation, vascularization, and re-epithelialization [19,22]. Modern dressings are derived from natural or synthetic polymers and classified into interactive, advanced interactive, and bioactive modern wound dressings [23,24]. Films and foams-based dressings come under semi-permeable interactive dressings, while hydrocolloids dressings, hydrogels dressings, smart wound dressings, electrospun nanofibrous wound dressings, antimicrobial wound dressings, and 3D printed wound dressings belong to advanced interactive dressings [1,17,25]. Furthermore, tissue-engineered skin equivalents and cell-based wound dressings belong to bioactive wound dressings [18,26,27]. Moreover, modern wound dressings are semi-occlusive or occlusive and stretch from hydrogel dressings, cell-based dressings, smart stimuli-responsive dressings, antimicrobial dressings to 3D printed dressings, etc. Moreover, modern wound dressings have been extensively employed for the treatment of various wounds in recent decades and are evaluated using a plethora of in vitro and in vivo studies. In order to develop and employ modern wound dressings to overcome the challenges in chronic wound management and improve outcomes of the healing process, there is a need to understand the detailed pathophysiology of wounds and the healing process, types of dressings, and features required in ideal dressings. Additionally, rationalized selection and execution of the in vitro and in vivo characterization and evaluation methods and assays is required to assess the suitability of modern wound dressings for improved healing.

This review provides an overview of the types of wounds and the wound healing process, the need for modern dressings, and their diverse types with advantages. Furthermore, in vitro and in vivo techniques employed to characterize and evaluate the suitability of modern dressings for wound healing are discussed in detail. Finally, the challenges associated with modern dressings and their future prospects have been discussed.

## 2. Wounds and the Wound Healing Process

### 2.1. Wounds and Pathophysiology of Wounds

Wounds are defined as skin/organ tissues with damaged integrity which is normally presented as cuts, punctures, burns, or other pathological conditions [28]. Wounds are classified into acute or chronic wounds based on the duration and nature of the wound-healing process [29]. Acute wounds are relatively healthy and result as a consequence of incisions, abrasions, puncture, avulsions, burns, and lacerations. Acute wounds heal rapidly and in an ordered manner within 8–12 weeks by proceeding through an intricate and organized wound-healing process using the cascade of cytokines, growth factors, and matrix proteins for restoration of skin’s structural and functional integrity [30]. On the contrary, chronic wounds heal much slower than acute wounds with approximately less than one-third wound closure in 12 weeks. Chronic wounds are considered hard to heal or non-healing wounds as these wounds do not follow a normal timely and ordered wound-healing process [31]. As per recent statistics, chronic non-healing wounds account for 2.5% of the total global population and manifest significant social and financial burden [9]. The structural composition of chronic wounds is complex and apart from the epidermal, dermal, and hypodermal layers, it also affects the deeper tissues such as bones and muscles. The occurrence of chronic wounds is attributed to the uncorrected status of wound healing due to the extended inflammation phase, abnormal immune response, persistent alternation in the wound microenvironment, cellular insufficiencies leading to a subdued proliferation phase, and an almost non-existent remodeling phase [32,33]. Chronic wounds are characterized by high inflammatory cytokines, elevated levels of matrix metalloproteinases, and a low mitogenic response, and the condition further gets complicated with poor vascularization, necrosis, neoplasia, and persistent trauma [31]. Apart from these, some other factors which extend the inflammatory phase and mediate eosinophil cationic protein destruction in case of chronic wounds include hormonal deficiencies, zinc deficiency, nutritional deficiency, and cold temperature [30]. These factors recruit neutrophils and macrophages to the wound site and result in an impaired wound healing process.

**Figure 1:**
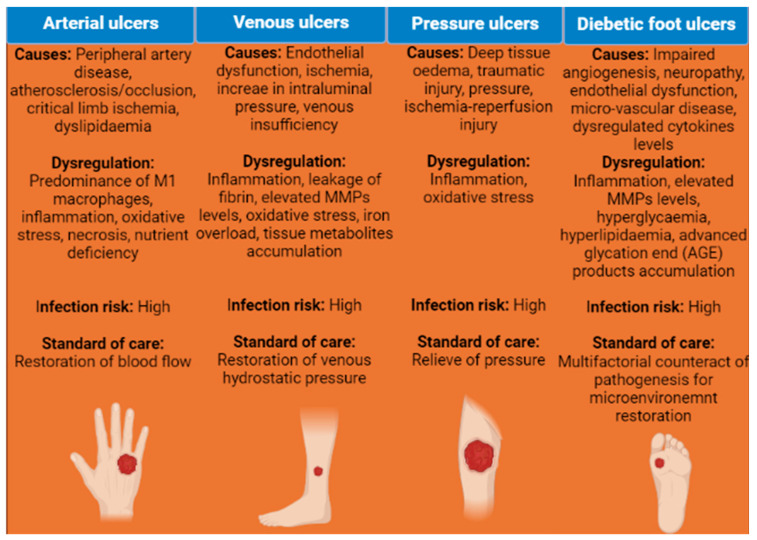
Etiology of different types of chronic wounds underlying causes, dysregulation, infection risk and standard of care.

The most common chronic wounds are associated with vascular insufficiency or metabolic diseases. Furthermore, on the basis of etiology, the wound healing society classified chronic wounds into four major types, namely, arterial ulcers, venous ulcers, pressure ulcers, and diabetic foot ulcers [32]. In addition, other types of chronic wounds include congenital vascular disorders (von-Willebrand disease, thrombocytopenia, and hemophilia), gangrenes, ischemia, and skin infections. Recent studies indicated a diabetic foot ulcers prevalence of 11.6% while venous and pressure ulcers affect 1–3% of the total population which are expected to rise in tandem with the aging population, low body mass index, and prolonged illness [34,35]. Arterial ulcers or peripheral artery disease (PAD) are associated with the occlusion of lower limb arteries, and advanced atherosclerosis (calcification and stenosis) and cause critical limb ischemia, tissue necrosis, and persistent inflammation [36]. The severity of chronic conditions of PAD increases with metabolic disease prevalence and is correlated to inflammation (for example, C-reactive protein) and fibrosis (for example, galectin-3) biomarkers [37,38]. Surgical bypass or endovascular treatment are the main therapeutic options for restoring the blood flow and revascularization in the lower extremity arteries (critical limb ischemia) to avoid amputation and promote the wound healing process [39]. Venous leg ulcers (VLUs) represent the most prevalent chronic wounds in the lower extremity of older patients with a recurrence rate of up to 70%. This type of ulcer results in damage of the superficial and deep venous system that progresses to venous insufficiency and 30–50% of patients fail to heal [40,41]. Venous ulcers are characterized by reduced blood flow, increased blood pressure, and the leakage of fibrin due to alteration of vessel wall permeability (endothelial dysfunction). The vascular dysfunction leads to excessive inflammation, increased levels of matrix metalloproteinases activity (MMP-1, 2, 9), redox imbalance (reactive oxygen and nitrogen species), reduced dispersion of oxygen, iron overload, shear stress, edema, and accumulation of tissue metabolites responsible for inadequate wound healing [41,42,43]. In another kind of a chronic wound, which normally occurs in old, paralyzed patients and people who are suffering from spinal cord injuries, deep tissue injuries limit blood flow to the surrounding tissue due to sustained stress and pressure in areas such as heels, sacrum, and shoulder blades. Furthermore, sustained mechanical loading due to shear pressure on the skin and underlying tissues results in oxidative stress, edema, persistent inflammation, decreased oxygen diffusion, and elevated levels of MMPs leading to necrosis and secondary tissue damage [44,45]. Once skin tissues are damaged with deep tissue injuries, it is extensive and irreversible. Negative pressure therapies and relieving the sustained pressure may help in promoting wound healing in this type of ulcer [46]. Diabetic foot ulcers (DFU) are the most common type of chronic wounds and affect approximately 15% of the global population. This type of chronic wound generally occurs in legs and feet and if not treated properly, there will be a need for limb amputation. DFU develops as a result of prolonged diabetic complications, viz., chronic systemic inflammation, hyperglycemia, ischemia, microangiopathy, oxidative stress, accumulation of advanced glycation end products (AGEs), neuropathy, and foot infections [47,48]. There is an initial delay in the inflammatory response after external injury due to ongoing chronic inflammation, maladaptive immune response, and neuropathy. Subsequently, progress to the proliferation phase is hindered once the inflammation phase of wound healing starts. This sustained inflammation and non-responsiveness of cellular mediators responsible for repair leads to impaired wound healing in case of DFU [47,49,50,51]. Currently, there are not any pharmacological treatments available for DFU owing to the complexity of the wound microenvironment. However, offloading of pressure, infection management, and debridement are recommended as standard care [52]. A summarized description of the etiology of different chronic wounds along with causes, infection risk, types of dysregulation, and standard treatment approaches are listed in Figure 1. Taken together, accurate diagnosis of underlying causes considering both exogenous and intrinsic confounding factors is crucial in chronic wound management owing to its given dysregulation complexity for acceleration of wound healing. Furthermore, restoration of cellular and molecular dysfunction by overcoming impaired endogenous wound repair mechanisms pave way for the exploration of newer and innovative therapeutic modalities for chronic wound treatment.

### 2.2. Wound Healing Process

**Figure 2:**
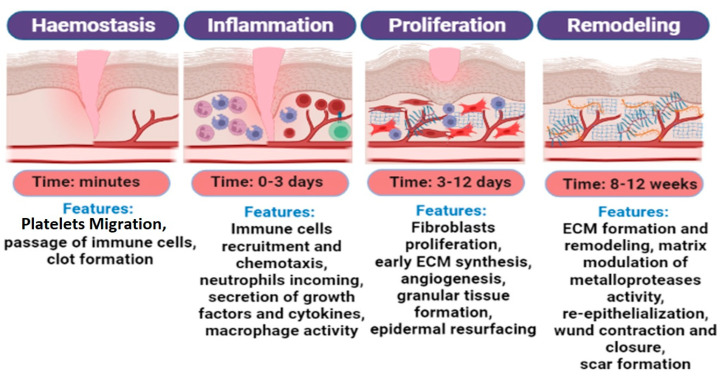
Different phases of wound healing process with their characteristic features.

Wound healing is a complex and intricate biological process, which initiates immediately after cutaneous damage to skin tissue in order to restore structural and functional skin tissue integrity [1,53]. The wound-healing process is affected by various factors, viz., patient age, degree of damage, and nature of the pathological process (autoimmune diseases, metabolic syndromes, vascular diseases) [4]. The healing process in general is mediated by a cascade of precisely orchestrated events that progresses through four distinct physiological phases hemostasis, inflammation, proliferation, and tissue remodeling involving cell migration and proliferation, growth factors action, and extracellular matrix (ECM) components modulation [54,55] (Figure 2). The first hemostasis phase of wound healing process starts immediately after injury to stop the bleeding. Herein, platelets arrive at the wound site and starts promoting coagulation by forming a fibrin clot to avoid microbial contamination and prevent further blood loss mediated through vasoconstriction [56]. Furthermore, the inflammatory phase starts almost simultaneously with hemostasis which are activated platelets secreted cytokines by recruiting first neutrophils followed by monocytes/macrophages and lymphocytes. These neutrophils help decontaminate the wound region through the secretion of antimicrobial peptides/proteases and production of reactive oxygen species (ROS) [57]. Neutrophils lie in the wounded region for 24 h before undergoing apoptosis. Thereafter, macrophages and lymphocytes secrete growth factors (vascular endothelial growth factor) and cytokines (interleukin-17), help in the removal of apoptotic neutrophils, and exert specific immune response against pathogens through B and T lymphocytes, respectively [56]. The secreted growth factors further stimulate the proliferation of fibroblasts, keratinocytes, and endothelial cells in order to start the next phase of the wound-healing process. Subsequently, the proliferation phase starts which lasts for 2–3 days and is characterized by granulation tissue formation, ECM components (hyaluronic acid, procollagen, proteoglycans, fibronectin) production, and neovascularization through migration and proliferation of skin cells. In this phase, the dominant cell-type, fibroblasts, migrate to the wound site and differentiate into myofibroblasts and produce ECM components [58]. Endothelial cells further migrate to the wound site and promote new blood vessel growth. On the other hand, keratinocytes migration and proliferation which are aided by plasmin through removal of excessive fibrin help in restoration of the protective epithelial barrier through re-epithelialization [56]. Moreover, ECM deposition and angiogenesis are promoted by crosstalk between all skin cells. In addition, secreted cytokines modulate the integrins present in macrophages and lead to phenotypical changes from pro-inflammatory phenotype (M1) type to the anti-inflammatory one (M2) type macrophages to ensure the resolution of inflammation for better wound healing. Thereafter, the last phase of wound healing is tissue remodeling wherein ECM remodeling occurs through modulation of MMPS activity [5,59,60]. In this final phase, deposition of collagen type III is replaced by collagen type I until restoration of normal skin ratio. As a result, tensile strength increases correlated with enhanced deposition of collagen. This is the maturation phase wherein wound contraction occurs and leads to wound closure and repair.

## 3. Wound Dressings and Recent Advancements

Wound dressings are devices utilized for protecting the damaged surface and promoting wound-healing process through direct interaction with the wounds, providing the moist environment, and adsorption of wound exudates [1]. Therefore, the selection of appropriate wound dressings depending on wound types and their pathophysiological conditions is a crucial requirement for quality wound healing. Wound dressings are in practice since ancient civilizations (2000 BC) as bandages and other traditional dressings to provide a protective covering to the wounds [61]. However, the gauze/bandages are now replaced with various advanced interactive types of wound dressings for the acceleration of the wound-healing process. Wound dressings provide an effective way for managing both acute and chronic wounds by creating a favorable microenvironment for preventing infection and improving wound healing. Recent advancements in materials science and technology and nanotechnology led to the development of a vast number of advanced wound dressings for the treatment of diverse kinds of wounds. Over the past decade, the development of a new futuristic class of wound dressings to monitor the wound healing progress and overall healing status smartly in real time presents an exciting approach for treatment and management of different wounds.

**Figure 3:**
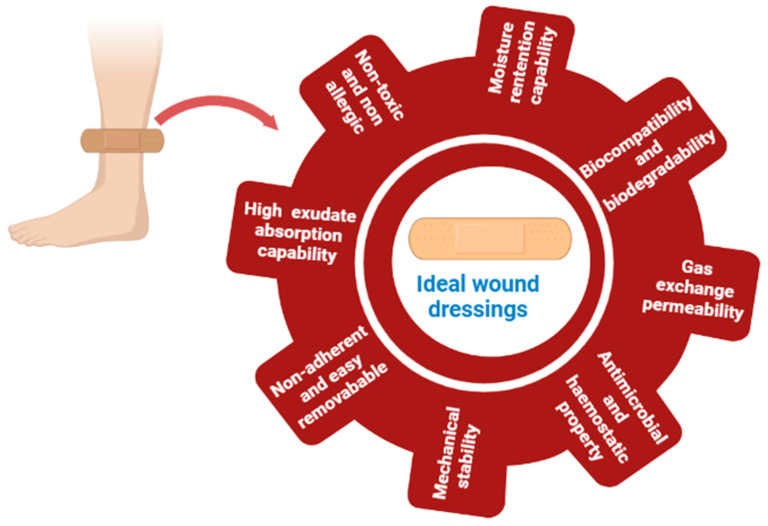
Prerequisite characteristics of ideal wound dressings.

Although an exact and univocal consensus about an ideal wound dressing is not present, there are certain characteristics that are pivotal for regeneration of damaged skin tissues (Figure 3). Wound dressings ensure optimal wound healing and should be used according to the wound type. Ideal wound dressings: (1) Should maintain a moist environment; (2) Allow gaseous exchange between wounded tissue and dressings; (3) Should adapt and conform to the wound surface; (4) Have the capability to absorb the high volume of wound exudates; (5) Should promote cell migration and proliferation (prerequisite for angiogenesis, migration of epidermal cells and tissue repair); (6) Exhibit a hemostatic action to prevent blood loss; (7) Should permit thermal insulation to maintain proper tissue temperature to improve the blood flow; (8) Have capability to prevent bacterial infection; (9) Should be occlusive and non-adherent; (10) Should be non-toxic, non-allergic, and biocompatible with good mechanical stability; (11) Should be easy to remove without damaging the skin; (12) Must allow debridement action to promote leucocytes migration and enzymes accumulation; (13) Should be able to minimize scar formation; (14) Should have easy availability and cost-effective [53,62,63].

Among all the required properties, the most important characteristic of ideal wound dressings is their moisture retention capability. The importance of moist environment maintenance requirement was highlighted earlier by Winter for epidermal healing and preventing wound maceration when dressings are exposed to air and dry conditions [64]. The wound-healing process is amplified many folds if applied wound dressings maintain the wound environment moist as it favors enzyme activity, epidermal growth factor functionality, and collagen deposition for the promotion of skin regeneration [65]. Another important criterion is gas (mainly oxygen exchange) permeability between the exterior and interior of the dressing. Another cardinal performance parameter of an ideal wound dressing includes its ability to manage wound exudate as its composition varies with different types of wounds [66]. The secreted wound exudate in acute wounds promotes tissue repair and remodeling, however, in the case of highly exuding chronic wounds the proliferation of wound reconstructing cells is slowed down due to the presence of elevated levels proteases and proinflammatory cytokines. The highly exudating chronic wounds promote bacterial growth, spreading that ultimately slows down the healing process [67]. Therefore, wound dressings in the case of chronic wounds should have proper liquid absorption in order to prevent fluid leakage through or around the dressing. The ideal wound dressing selection is conducted based on the secreted exudate properties, viz., size, type, volume, and viscosity selection, for chronic wounds [68]. Furthermore, the biocompatibility, biodegradability, and mechanical properties of ideal wound dressings materials have paramount importance in wound healing. To attempt this, biocompatible and biodegradable wound dressings should be utilized to match the healing rate with the degradation rate of dressings materials [69]. Wound dressings should provide support against skin mechanical stresses (linear and shear stresses) to minimize patient discomfort and ensure the ability to cater different types of wounds [70,71].

### 3.1. Traditional Wound Dressings and Their Limitations

Currently, several types of wound dressings are clinically available which are selected based on physical, chemical, and biological properties for treatment of different types of wounds such as burns, ulcers, and skin disorders. Based on their properties, wound dressings are further classified into traditional and modern wound dressings. Furthermore, based on the interactions between dressing and the wound environment, wound dressings are again categorized into passive, interactive, and bioactive dressings [72]. Traditional wound dressings as sterilized gauze/bandages for surgical dressings have been around since 1970 and before for utilization in wound management. To date, the most developed classic wound dressings forms include gauze/bandages, foam/sponge, and others (cotton wool, tulle, silver sulfadiazine, plasters, etc.) with specific properties to aid in wound healing process [73,74,75]. The traditional wound dressings are considered passive or non-occlusive dressing as these dressings facilitates material transfer (gas and moisture molecules) between dressings and environment unlike interactive or occlusive dressings [72]. Gauzes and bandages represent dry wound dressings and are utilized for wound healing owing to their hygroscopic nature, high affinity for skin, and biocompatibility [76,77]. Examples of gauze dressings include cotton fibers, viscose, and polyesters in both woven and non-woven forms to apply on dry and well-cleaned wounds. Cotton gauze is the most widely used earlier for wound healing, but its usage is limited nowadays due to its inert nature leading to limited wound healing. In order to overcome this limitation, cotton gauges are modified with functional components such as antimicrobials to promote wound healing. Bandages are utilized as secondary wound dressings and mainly comprise cellulose, cotton, wool, polyester, rayon, or polyamide. Cotton bandages are used for cleaning wounds but stick to wound surfaces due to fibrous nature. Therefore, cotton bandages are mostly utilized for dry arterial and venous ulcers. Other bandages made up of rayon, polyester, and polyamide bandages are non-adherent in nature and exhibit high water vapor permeability, which is suitable for treatment of granulated wounds with a mild to moderate exudate [19]. Conventional pharmaceutical preparations used with conventional dressings include paste, creams, beads, and ointments for wound healing application. All the traditional wound dressings are categorized as dry dressings and face their own limitations despite providing hemostatic properties, protecting covering, preventing infections, removing wound exudates, and being cost effective. Traditional wound dressings dry the wounds quickly attributed to their high wound exudates absorbent capacity that promotes bacterial growth and further spreading. Other disadvantages of passive dressings include adherent nature, frequent need to change dressings and materials transport capability in chronic wounds with high secreted wound exudates. The detachment of such wound dressings causes severe pain and damage that further limits their utilization in clinical practices [78]. In addition, these dressings may generate clots due to absorption of blood and exudates that do not come off easily and result in skin damage after removal. As traditional dressings are passive dressings, these are suitable for mainly dry wounds due to the lack of interaction between dressings and wound environment. Taken together, these disadvantages restrict the usage of traditional wound dressings and need replacement with modern wound dressings with improved properties.

### 3.2. Modern Wound Dressings

Wound healing is a dynamic process that progresses in different stages with specific molecular and physiological events, as discussed earlier. Therefore, different types of wound dressings are required at different stages of the wound healing process. Traditional wound dressings provide a protective covering to protect from external harm, however, these dressings cannot provide a moist environment, enough drainage of the wounds, and effective wound closure. Other limitations include adherence to wounds, frequent change requirements, and severe pain after removal. Therefore, to overcome the limitations presented by traditional wound dressings, much advanced modern wound dressings which initiatively facilitate wound closure and promote healing are developed and explored in recent decades. Modern dressings are semi-permeable and highly absorbent dressings that facilitate wound functions, accelerate granulation tissue formation, and promote cell proliferation and migration for rapid wound closure and repair in addition to providing moist environment [22]. Moreover, traditional wound dressings have now been replaced with modern dressings with advancements in materials science and technology, microfabrication technologies and nanotechnology. Over the years, numerous types of modern wound dressings have been developed to promote, diagnose, and monitor wound-healing process using a wide range of biomaterials [79]. Modern wound dressings are classified into three types i.e., interactive, advanced interactive, and bioactive wound dressings, based on their distinctive properties. Interactive wound dressings include semipermeable films, and foams, while advanced interactive dressings include polymeric wound dressings, nanofibrous wound dressings, hydrogels dressings, and hydrocolloid dressings. The third category includes the most advanced type of dressings termed as bioactive wound dressings. The example of bioactive wound dressings include tissue-engineered (bioengineered) skin equivalents, cell-based dressings, antimicrobial dressings, nanoparticles-loaded wound dressings, growth factors/cytokines/nanoparticles loaded wound dressings, stimuli-responsive smart wound dressings, biomechanical smart wound dressings, self-healing smart wound dressings, self-removal smart wound dressings, 3D bioprinted dressings and others [63,73]. Moreover, there is a dynamic interaction between the dressings and wound environment while using interactive, advanced interactive, and bioactive wound dressings for accelerated wound healing process [80]. In addition, the occlusive environment produced by interactive dressings provides moist environment and decreases bacterial infection risk [81]. A representative list of different types of modern dressings being employed for wound healing are displayed in Table 1.

The foremost requirement of wound dressings is to maintain and control moist environment that facilitates wound healing. The interactive wound dressings are also termed as moist wound dressings and presented by semipermeable films-based dressings, foams-based dressings, hydrogels dressings, nanofibrous dressings, hydrocolloids dressings and polymeric dressings produced by a wide range of polymers [126,127]. Film-based dressings are transparent and are semipermeable adhesives that easily conform to the wound’s surface. The advantages of using film-based dressings (such aspolyurethane films) include transparent nature for wound monitoring, O_2_ and CO_2_ gas exchange availability, impermeability to bacteria, high vapor transmission rate, and automatic scab removal ability. vary in size and thickness [128]. Initially, nylon films-based dressings with adhesive polyurethane edges for wound healing but they were unable to retain high volume of wound exudates and provided undesired outcomes, viz., skin maceration, bacterial proliferation, and liquid accumulation with the necessity to change frequently [129]. Thereafter, nylon film-based dressings were replaced with the most commonly utilized polyurethane-based semi-permeable films to overcome the associated limitations. These dressings are adhesive, adapt well to the wound, and retain a large volume of exudates. In addition, being transparent these dressings allow wound assessment with reduced pain by limiting frequent dressings changes requirement. These properties of polyurethane-based semi-permeable films promote re-epithelialization and wound healing [1]. Consequently, films derived from diverse polymeric biomaterials (natural and synthetic) have been utilized for healing superficial wounds, abrasions, infected wounds, surgical wounds, burns. The commercially available film-based dressings based on their adhesiveness, conformability and permeability include FDA-approved Hydrofilm/Hydrofilm™, Tegaderm™, Opsite™, and Biooclusive™ [130].

Furthermore, semi-permeable foams/sponges with highly connected porous structures have been applied to facilitate wound healing as well as hemostasis and wound exudates absorption. For example, polyurethane foams with antimicrobial and anti-inflammatory properties promoted re-epithelialization [131,132]. These dressings with good water vapor permeability are utilized in the treatment of ulcers of the lower limbs, pressure ulcers (stage I–IV), skin transplants, and mild burns (stage I–II) [133]. The commercially available dressings based on polyurethane include Allevyn™, Askina Foam™, Tielle™, and Lyofoam™ [90]. However, unsuitability for dry wounds and frequent changes limits the effectiveness of such dressings.

Hydrocolloids based wound dressings are the combination of elastomers, adhesive coatings, and gel-forming agents (pectin, alginate, carboxymethylcellulose, and gelatin) [22]. Hydrocolloid wound dressings belong to advanced interactive occlusive dressings and mainly comprise two layers. The outer layer is made up of a polyurethane layer which is impermeable to bacteria and the inner one is presented by a colloidal suspension that helps maintain a moist environment [134,135]. These dressings form a gel phase on the wound surface which helps in moisture retention and protection of granulation tissue through absorption of wound exudates. Hydrocolloid-based dressings are suitable for wounds with low to medium exudate secretion such as abrasions, abdominal incisions, burns, pressure ulcers, venous insufficiency ulcers, and neurosurgical wounds, and provide support to wound surface for up to 7 days [136]. Examples of commercially available hydrocolloid-based wound dressings include Comfeel™, and Tegasorb™, and Granuflex™ [137]. Nevertheless, these dressings are not appropriate for chronic with abundant wound exudates (such asneurotrophic ulcers) [138]. In addition, hydrocolloid wound dressings gel coatings adhere to the wound bed and are difficult to remove [139].

Moist wound dressings based on hydrogels have attained much attention in overcoming shortcomings of conventional wound dressings due to their moisture-retaining capability, high exudate absorption capacity and ability to promote migration, and proliferation of fibroblasts and keratinocytes [140,141,142]. Hydrogel-based wound dressings present the most promising advanced interactive dressings for active intervention in the wound healing process owing to their advantageous properties for wound care. The properties suitable for quality wound healing include maintenance of a moist environment, the high absorbent ability for wound exudates, adhesion-free wounds covering, and pain reduction through cooling effect [143]. Hydrogel-based dressings are made from either natural or synthetic polymers and comprise 90 wt.% of water and can absorb up to 1000 fold wound exudates for enhancement of cellular wound resolution and debridement [144]. As a consequence, hydrogel-based wound dressings are suitable for treatment of dry wounds, necrotic wounds, wounds with medium to moderate exudates, deep wounds, fistulas, surface wounds, and burns. A variety of hydrogel-based wound dressings have been developed with different properties such as self-healing, antibacterial ability, drug release property, adhesive property, injectability, and others for effective wound healing [145,146,147]. However, limited mechanical properties impede clinical implementation of hydrogel-based wound dressings and need further improvements.

Polymeric wound dressings derived from either natural, synthetic, or semisynthetic polymers present a prospective ideal delivery platform for the controlled delivery of antibiotics, drugs, growth factors, and other therapeutic agents in the proximity of the wounded area for improved wound healing. Polymeric dressings derived from natural biomaterials include chitosan, silk, alginate, cellulose, hyaluronic acid, dextran, pectin, and pullulan, while synthetic polymers-based dressings include mainly poly (vinyl alcohol), poly-ε-caprolactone, polyurethane, and poly(lactide-co-glycolide). The advantages of polymer-based dressings include biocompatibility, biodegradability, functionalization ability, mechanical stability, and integrity. Among polymeric wound dressings, alginate dressings comprising of alginate and calcium salts form gel on the wound surface and thus suitable for heavy drainage wounds owing to its ions exchangeability with the native skin tissues [148]. Alginate-based wound dressings materials such as films, hydrocolloids, fibers, foams, and hydrogels have been applied for the treatment of infected wounds, ulcers, fistulas, blooding wounds, severe burns, surgical wounds etc. Owing to gel-forming ability, highly absorbent alginate-based dressing materials are applied on both infected and non-infected wounds as they can absorb up to 20 times fluid than their original weight. These dressings retain a moist environment and dressings particularly functionalized with antibacterial agents are more prominent. Despite of its promising properties, skin dryness and a burning sensation occur due to integration between highly absorbent alginate wound dressings and wound bed fluid [149]. Therefore, these dressings should not be applied on dry wounds as increased pressure may impair the wound healing process and causes necrosis.

Bioactive wound dressings are wound dressings incorporated with bioactive molecules such as bioengineered living skin equivalents, conducting wound dressings, cell-based wound dressings, antimicrobial dressings, 3D bioprinted wound dressings, etc. In recent years, with the development in materials science and technology, bioactive wound dressings are upgraded to cell-based dressings to address the need for complex and biomimetic therapies to modulate protective properties, moisture retention ability, pharmacological, and structural properties. The advantage of cell-based dressings lies in the capability to incorporate and/or deliver biological molecules (growth factors, ECM components, or cytokines), fibroblasts, keratinocytes, macrophages, and mesenchymal stem cells (MSCs) [18,150]. Moreover, cell-based strategies are employed in the wound dressings domain to promote chronic wound regeneration by normalizing the impaired intercellular communication and reducing the continuous inflammatory state. Cell-based dressings therapies are categorized into three types, viz., bioengineered living skin equivalents, stem cells-based dressings, and other cells-based dressings (macrophages). Bioengineered living skin equivalents or tissue-engineered skin equivalents are the most common bioactive wound dressings comprising skin cells (fibroblasts, keratinocytes, melanocytes) and natural/synthetic polymer-derived biomaterials in various forms (films. hydrogels, scaffolds, nanofibers etc.). The natural polymeric biomaterials being utilized for fabricating bioengineered skin equivalents include collagen, silk, chitosan, hyaluronic acid, and others. In addition, various biocompatible synthetic polymer-based biomaterials are also employed. Bioengineered living skin equivalents can be further categorized into first-generation and second-generation wound dressings. First generation wound dressings include mono/bilayer skin equivalents, while second-generation dressings are presented by bi/multilayered skin equivalents. Examples of commercially available bioengineered skin equivalents are ApliGraf^®^ (collagen-based), Dermagraft^®^ (polyglactin-based), TransCyte^®^ (nylon mesh based), Integra^®^, AlloMax^®^, AlloDerm^®^, GraftJacket^®^ (acellular dermal matrix) etc. These bioactive wound dressings have been utilized for treatment of non-infected diabetic neuropathic ulcers (full-thickness), severe burns, deep wounds with loss of tissue, venous ulcers (partial and full-thickness), as well as diabetic foot ulcers [151]. The tissue engineered skin equivalents are extended to cell-based wound dressings containing mesenchymal stem cells for wound healing. Furthermore, stem cells-based dressings and other cells-based dressings can be fabricated in different formats, viz., scaffolds, hydrogels, nanofibers, 3D printed dressings. Numerous studies have been conducted using stem cells-based dressings, particularly MSCs and reached to clinical trials studies (Table 2).

Another prominent bioactive wound dressing includes smart dressings incorporated with bioactive compounds to deliver at the wound site to enhance the interactions between the wound environment and dressings for improved wound healing [158]. Smart dressings perform various functions (1) respond to physical/chemical changes of wounds via integration with stimuli-responsive materials, (2) accelerates wound closure through biomechanical character, (3) endure motion/tensile strength through self-healing property, (4) ease in dressings removal through self-removal capability, and (5) timely report of wounds status by real-time monitoring [73].

**Figure 4:**
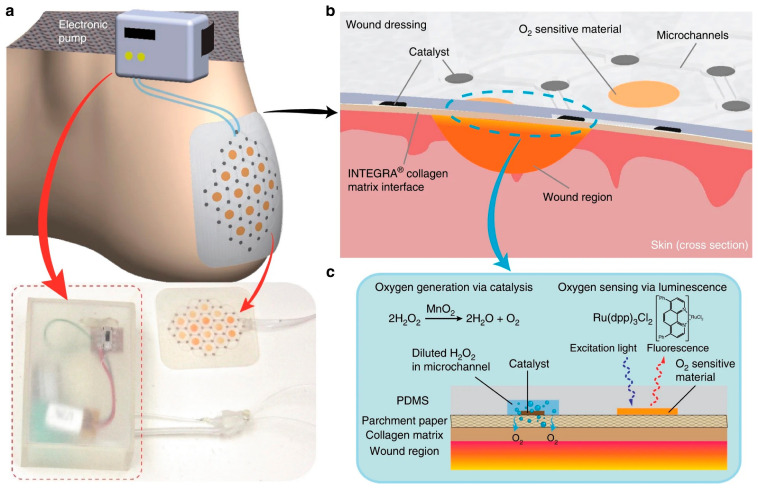
Smart stimuli responsive wound dressings. (**a**) Schematic illustration of smart stimuli responsive wound patch for foot ulcers treatment, (**b**) oxygen generation and sensing through smart wound patch in wound area, and (**c**) mechanism of oxygen generation for sensing using flexible smart wound dressings. Reproduced from ref. [72] with permission from Nature Publishing Group. This work is licensed under a Creative Commons Attribution 4.0 (CC BY) International License.

Over the years, numerous smart dressings have been developed based on the smart properties, viz., stimuli-responsive (pH, temperature, oxygen, glucose, etc.) wound dressing, self-healing wound dressing, biomechanical wound dressing, and self-removable wound dressing for moving wounds for treatment of diverse types of wounds depending on the functionalization [115,116,117,118,119,120]. A flexible smart wound dressing with oxygen-sensing ability for treatment of foot ulcers is shown in Figure 4. Recently, the development of innovative wearable smart wound dressing further advanced the concept of smart bioactive dressing for wound healing applications [159]. Moreover, the advancements in modern dressings over the years with the development of various emerging interactive and bioactive wound dressings have shown promising results and have pushed the forefronts in wound healing domain.

## 4. In Vitro Characterization Methods for Wound Dressing Materials

Currently, numerous versatile modern wound dressings made from natural, synthetic, or semisynthetic biomaterials with varying physicochemical properties are available for the treatment of acute and chronic wounds. Therefore, a thorough characterization of developed wound dressings are pertinent in order to utilize these wound dressings for specific wound healing applications. In this regard, physicochemical and in vitro biological characterization of wound dressings are carried out using different methods to determine/evaluate the properties of wound dressings such as surface structure, moisture retention ability, capability to absorb fluids, chemical composition, flexibility, mechanical properties, degradation properties, antimicrobial properties, drug release pattern, cytocompatibility, healing ability, and others. This section describes the various in vitro physicochemical characterization methods currently being employed for the characterization of wound dressings.

### 4.1. In Vitro Methods for Characterization of Wound Dressing

#### 4.1.1. Thickness Measurement

The thickness of wound dressings ensures uniform drug distribution and mechanical properties [160]. There are various parameters that affect the dressing’s thickness, viz., fabrication method, amount/volume of casting gel/solution and drying surface flatness [161]. dressing’s thickness is measured in millimeters (mm) and generally determined by taking random measurements (n = 5–10) using digital micrometer, caliper, or manual screw gauge and scanning electron microscopic (SEM) images. The thickness is represented by taking average of the noted random measurements [162,163,164,165].

#### 4.1.2. Water Vapor Transmission Rate/Moisture Transport Studies

The water vapor transmission rate (WVTR) denotes one of the basic physical property of wound dressings as it helps regulate the moist environment, assess the moisture transfer and exudate absorption rate along with controlling water loss during the wound healing process [166,167]. Moreover, as discussed above, ideal wound dressings should also allow moisture transport (for efficient gas exchange) apart from good wound exudates absorption ability for the wound healing process. In an earlier studies, the value of WVTR in the range of 100 to 3300 g/m^2^/24 h is found suitable for commercially available wound dressings to maintain the optimum moisture content, for the promotion of cell proliferation and functions without causing dehydration [160]. However, different types of wounds would have different wound dressings requirements with different WVTR. If WVTR is extremely high it may lead to dehydration, while less than optimum WVTR generates excessive wound exudates and thus rise the risk for infections [127]. Therefore, the determination and maintenance of optimum WVTR of different wound dressings is pivotal for wound healing applications. The WVTR of wound dressings are evaluated according to the ASTM E96 procedure using either the desiccant method or the water method as per requirement [166,168].

#### 4.1.3. Swelling/Expansion Studies

Swelling ability is one of the important parameters of wound dressings as it helps to absorb excess wound exudate through expansion, maintain a moist environment, aid in wound debridement, facilitates nutrients diffusion of signaling molecules and allows the release of incorporated drugs to wound bed [169,170]. The swelling ability of wound dressings is represented by the swelling ratio, swelling index, or swelling rate. Several factors influence the swelling rate, viz., physicochemical properties of utilized biomaterials, presence of hydrophilic groups, crosslinking agents, crosslinking density etc. [171]. Researchers have reported various methods for the determination of swelling index of wound dressings such as estimation of diameter expansion of circular dressings and gravimetric method as function of time at predetermined time intervals [160,172]. In gravimetric method, dried wound dressings are immersed in either distilled water or phosphate-buffered saline (PBS) solution (pH = 7.4 at 37 °C). PBS mimics the physiological conditions of wound exudate [173]. The weight of swollen dressings is recorded at predetermined time intervals until equilibrium is reached. The swelling index is calculated as an increase in weight of dressings after immersion in swelling medium as percentage or ratio.

#### 4.1.4. Instrumental Characterization

The physicochemical properties of wound dressings are determined by using various instrumental techniques such as scanning electron microscopy (SEM), X-ray diffraction (XRD), thermogravimetric analysis (TGA), differential scanning calorimetry (DSC), Fourier transform infrared spectroscopy (FTIR), high performance liquid chromatography (HPLC), UV-visible spectroscopy, nuclear magnetic resonance (NMR), atomic force microscopy (AFM), etc. [92,174,175,176]. SEM is utilized for analyzing surface morphology and porosity of dressing materials, while surface roughness is determined by AFM. The chemical composition and functional groups present in biomaterials are determined by FTIR and NMR [177]. In addition, FTIR confirm the purity of the material and interactions between different functional groups. Furthermore, the thermal properties (mass loss and thermal stability) of wound dressings are determined by TGA and DSC. The physical form of the biomaterials and crystallinity are measured by XRD while HPLC and UV-spectroscopy help in the determination of content uniformity through the assessment of the active substance present within the dressing materials.

#### 4.1.5. Mechanical Characterization

The healing of different types of wounds with varying etiology and anatomical locations needs specific mechanical properties to regulate the healing process. Mechanical (compressive and tensile) properties of dressings affect cell attachment, migration and proliferation which are prerequisites for wound healing [178]. Mechanical properties of dressings vary with polymer type, volume and concentration of polymer utilized and presence of hybrid/composite structure. Wound dressings with suitable mechanical properties provide essential physical integrity for the promotion of wound healing with less or no scarring. The mechanical properties of dressings are represented by compression strength and modulus (Young’s modulus), tensile strength, and percent elongation at break. The percent elongation at break is defined as the point wherein materials will break after a sufficient increase in length. Tensile strength and percent elongation at break of film/nanofibers-based dressings is determined by Universal Testing Machine (UTM) or a texture analyzer according to the Active Standard Test Method (ASTM) D882 method [92,179]. The values of tensile strength and elongation at break should be in the range of 2.5–16 N/mm^2^ (MPa) and 70% in order to resist natural deformation, respectively [160]. On the other hand, compressive properties (Young’s elastic modulus) of scaffold/hydrogels/3D printed based wound dressings are determined by UTM and AFM according to ASTM guidelines [180,181]. Young’s modulus is defined as ratio of stress by strain and generally directly proportional to the slope of the stress-strain curve. The Young’s modulus of skin tissue lies in the range of 4.6 to 20 Mpa [182]. Therefore, wound dressings should be fabricated in this range in order to be utilized for wound healing.

The wound dressings should not only withstand the force/stress/stretching exerted by the skin or other body parts but it should deal with the variations in the outer environmental conditions [183]. Therefore, in addition to above mentioned mechanical tests, the dynamic rheological/mechanical analysis (viscoelastic properties) of certain types of wound dressing materials (such ashydrogels) are determined using parallel palate rotational rheometers [183,184]. The viscoelastic properties also provide information about the internal network structure of the dressing material. In this case, the frequency sweep test is most commonly performed by the researchers in which circular pieces of the dressing material (hydrogels/films) are placed between the parallel plates of the rheometer and storage (G′) and loss (G″) moduli are determined over the frequency range (mostly 0.1 to 100 Hz) at constant strain (within linear viscoelastic range as pre-determined by strain sweep test) and temperature (preferably human body temperature i.e., 37 °C) [184,185,186,187,188]. The value of G′ provides information about the dressing materials’ ability to return to their original position when the deformation force (stress) is removed (indicates materials’ stiffness) while the value of G″ provides information about energy lost after each deformation cycle (indicates materials’ elasticity) [186]. For hydrogel-based dressing materials, the value of the G′ is greater than G″ indicating the stable gel structure (network) while if G″ is greater than G′ with increasing strain, it indicates destruction of the hydrogel network [183,184,185,188]. Additionally, the viscoelastic properties of the hydrogel-based dressings can also be investigated at different temperatures (to investigate the effect of temperature on G′ and G″), by using hydrogels sample samples with different amounts of water/simulated wound fluid (to understand the effect of different amount of exudates present in dressing on the on G′ and G″) and to determine the adhesive performances [186,187]. Previously, numerous researchers has reported the dynamic rheological analysis of hydrogels-based wound dressing to investigate their mechanical characters and structural properties [183,184,185,186,187].

#### 4.1.6. In Vitro Antimicrobial Studies

The wound healing process is delayed by bacterial infections of wounds and if not controlled may prove to be life-threatening to patients. Consequently, wound dressings with strong antimicrobial properties are vital to prevent bacterial infections and promote efficient wound healing process [189]. A variety of polymers with inherent antimicrobial property such as chitosan, methacrylated polymers, and polymers containing silver nanoparticles have been utilized for the fabrication of wound dressings to prevent bacterial infections. More recently, antimicrobial dressings incorporating antimicrobial agents have been developed as alternative viable options to reduce the bacterial load and improve the wound healing process [25].

Several methods are currently being employed for determining the antimicrobial properties of dressings such as the determination of zone of inhibition (ZOI) using disc-and well-diffusion methods while agar, and broth dilution methods are used to determine minimum inhibitory concentration (MIC) [174,190,191,192]. Mostly, the disc diffusion method is used to estimate the antimicrobial properties of the dressing materials or antibacterial agent loaded dressing [160,161,167,170,177,192,193,194]. Briefly, in this method, the dressing materials (hydrogel, films, sponge, etc.) are cut into disc-shaped pieces and placed on the agar plates that are inoculated with the bacterial strain (mostly *S. aureus* and *E. coli*). The plates are then incubated for a certain period of time and the diameters of the ZOI are determined to estimate the antibacterial effect of the dressings [192,194]. The standard antibiotic discs (commercially available) or filter paper discs impregnated with antibacterial agent solutions of antibiotic/antimicrobial agent are used as control positive while blank dressing materials and blank discs are used as a negative control [193]. Alternatively, in the well-diffusion method, wells of a constant width are created in agar plates inoculated with microbial strains, dressing material/antibacterial agents are filled in these wells, and ZOI are determined after incubation for different time-periods [195].

On the other hand, agar or broth dilution methods are used to estimate the MIC of the antimicrobial agents. In the broth dilution method, liquid media (inoculated with a definite number of bacteria) is treated with serial dilutions antimicrobial agents while in the agar diffusion method, different concentrations of the antimicrobial agents are incorporated in the agar (solid media) and the definite number of the bacterial are inoculated on these plates [196]. In both these methods, the MIC is the lowest concentration of antibacterial/antimicrobial agents that stops the visible growth of bacteria/microbes. However, the MIC of the wound dressing is not commonly investigated and these tests can be used to investigate the MIC of the polymers or particles (nano or micro) used in dressing materials, and antimicrobial agents loaded in the dressing [190]. Another method that is commonly used to investigate the antibacterial/antimicrobial properties of the dressings is the colony count or colony forming unit (CFU) method. In this method, wound dressing materials are incubated with the broth media containing a definite number of bacteria for a defined time period and after incubation, the number of remaining bacteria is counted and the inhibitory effect (percentage) or antibacterial ratio is calculated in comparison with untreated bacteria [192,193,197].

#### 4.1.7. Methods of Drug Loading and Release Studies

Wound dressing with drug release ability can deliver drugs/therapeutic agents in controlled and sustained manner for promoting wound healing and preventing bacterial infections [160]. Wound dressing types such as films, foams, hydrogels, and nanofibrous forms have been utilized for drug loading and delivery. The most effective drug delivery cargos for wound healing include hydrogel-based, nanoparticle containing and foam-based dressings as they provide controlled and sustained drug release for longer time than film-based dressings [191]. These dressings can be loaded with different types of the therapeutic agent/drug such aa the drugs that promote wound healing or prevent wound infections (antibiotics) [161,198]. The entrapment efficiency (the percent of the initial amount of drug added into the formulation that is entrapped/loaded) into these dressing materials is estimated using different techniques that depends on the type, and preparation method of the dressings. For example, in polymeric blended film dressings the desired concentration of the drug that is intended to be incorporated Into the dressing are added into the solution/gel used to cast the films [161]. In this method the entire drug added into casting gels is present in the final formulation (dried films) and calculation of the loading or entrapment efficiency is not required. On the other hand, for dressings based on swellable materials (such ashydrogel-based dressing), mostly the drugs are loaded into the dressing by the swelling diffusion method in which a weighted amount of the dressing material is immersed into the drug solution of the known concentration and allowed to swell for a defined time, then the dressing material is removed and dried [199,200]. The drug entrapped into the dressing material is calculated in terms of entrapment efficiency by quantifying the amount of the drug present in the residual drug loading solution using suitable quantification techniques (spectrophotometer, HPLC, etc.) and entrapment efficiency (EE%) is calculated using following equation.

$$EE\;\left(\%\right)=\frac{\mathrm{amount}\;\mathrm{of}\;\mathrm{drug}\;\mathrm{in}\;\mathrm{loading}\;\mathrm{solution}\;-\mathrm{amount}\;\mathrm{of}\;\mathrm{drug}\;\mathrm{in}\;\mathrm{residual}\;\mathrm{solution}}{\mathrm{amount}\;\mathrm{of}\;\mathrm{drug}\;\mathrm{in}\;\mathrm{loading}\;\mathrm{solution}}\;\times 100$$

In some dressing materials (electrospun, 3D composites, patches and hydrogels) known amount of drug is added into the solution used for preparation of these dressings but the entire preparation solution is not converted into the dressing and the drug may be lost during preparation steps. For such dressing materials, drug EE can be estimated by dissolving known weight of the drug-loaded dressing in suitable solvent, quantifying the total amount of drug present using appropriate assay technique and calculating EE (%) using the equation [201,202,203,204].

$$EE\;\left(\%\right)=\frac{Measured\;loaded\;amount\;of\;drug}{\mathrm{Theoratical}\;\mathrm{amount}\;\mathrm{of}\;\mathrm{drug}\;\mathrm{added}\;\mathrm{in}\;\mathrm{the}\;\mathrm{prepeation}\;\mathrm{solution}}\times 100$$

The loading efficiency (LE%) of the drug in the dressing material is used to express the amount of the drug that is loaded per weight of the dressing materials and is calculated using following equation [203,205].

(1) $$LE\;\left(\%\right)=\frac{Amount\;of\;drug\;loaded}{\mathrm{Weight}\;\mathrm{of}\;\mathrm{the}\;\mathrm{hydrogels}}\times 100$$

The amount of drug release from dressings is evaluated by measuring the cumulative release of drugs over specified time intervals. The methods used to evaluate the drug permeation and drug release ability of dressings includes in vitro dissolution study and Franz Diffusion Test system [206,207]. In the most common method in vitro dissolution method, in which weighed amount of the drug-loaded dressing is placed in the specified volume of the release/dissolution media (the volume should be sufficient to maintain sink conditions). The different medium utilized for drug release include deionized water, PBS (pH = 7.4/6.8), acetate buffer (pH = 5.5), a mixture of ethanol/PBS, and simulated exudate fluid (SEF), [162,191,208,209,210,211,212]. Among all these release media, SEF comprising sodium (142 mM) and calcium ions (2.5 mM) is most relevant as it mimics the wound fluid. The experiment can be performed in a beaker or is dissolution media depending upon the dressings. In so dressing (such asnanomaterials-loaded dressings) the dialysis membrane bags can be used place the dressing material then these bags are immersed in the release media. The samples are collected from the release media at definite periods of time and the amount of the drug released is quantified in these samples. There are various quantification techniques (instruments) that can be employed to determine the amount of drug released in media based on sample absorption values calculated using a predetermined calibration curve. These instruments include UV–vis spectrometer, flame atomic absorption spectroscopy (FAAS), HPLC, and plasma atomic emission spectroscopy [182,208,212,213]. The cumulative release (%) at sampling time is calculated dividing the amount of drug released until sampling time with total amount of loaded drug. Another method to determine drug release and permeation of an active ingredient includes Franz diffusion cell (FDC) system [160,161]. The FDC system comprises two chambers separated by a synthetic membrane or excised skin (lab animals or human) at constant temperature of 37 ± 1 °C. The upper chamber (donor compartment) contain drug-loaded wound dressings and the lower chamber contains release media, viz., PBS [207]. The drug dissolves and permeates through the separating membrane and is present in the lower compartment from where known volume of the samples are taken a different time intervals and cumulative amount drug permeated is calculated as a function of time.

## 5. In Vitro Cell-Culture/Wound Healing Models

### 5.1. Commonly Used Cell Lines and Rationale of Using

In order improve the healing outcomes of the wound dressing, a wide array of cell types has been utilized to evaluate the evaluated wound dressings. The important cell types extensively utilized in in vitro studies include fibroblasts, keratinocytes, vascular endothelial cells, melanocytes, pericytes, and stem cells [214,215,216,217]. Fibroblasts and keratinocytes are the most commonly employed cells in in vitro cell culture studies as they are progenitor cells of the skin and integral component of the dermis and epidermis skin layers, respectively. Furthermore, vascular endothelial cells have been utilized to promote vascularization for improving wound healing. Other cell types, pericytes and, melanocytes are utilized along with progenitor skin cells to recapitulate the fully functional skin tissue. In recent years, stem cell-based wound healing approaches have garnered much attention due to their ease of isolation, self-renewal ability and differentiation ability into multiple cell types using specific growth factors for the induction of physiological functions and skin tissue regeneration. Moreover, stem cells prevent wound contracture and scar formation, mediate rapid wound closure, and accelerate wound healing and skin regeneration along with the formation of skin appendages, unlike other cell types. Numerous adult stem cell sources such as bone marrow, adipose, Wharton jelly, epidermal stem cells and emerging induced pluripotent stem cells are utilized in in vitro evaluation of wound dressings [218,219,220]. In addition, stem cells derived exosomes have demonstrated accelerated wound healing as it’s a reservoir of a wide range of growth factors that are essential for wound healing [221].

### 5.2. Cytotoxicity Assessment

In vitro cytotoxicity assessment of wound dressing materials is monitored by inhibition of cell growth (cell viability measurement) supported by evaluation of cell morphology using diverse skin cell lines such as human dermal fibroblasts, murine fibroblasts, epidermal stem cells, human adult keratinocytes, and various stem cell sources. Numerous methods can be used to test the effect of the drug molecules, drug delivery carries and biomaterials (such asdressings) on the viability of the cells. These cell toxicity assays can be classified as dye exclusion (trypan blue), metabolic activity (MTT, MTS, XTT), cell metabolism (alamrBlue, Calcein AM), ATP, clonogenic cell survival, sulforhodamine B, protease viability marker, DNA synthesis and Raman micro-spectroscopy assays. The principles, advantages and disadvantages of these cytotoxicity and proliferation assays have be comprehensively reviewed by [222]. Among these assay methods, 3-(4,5-dimethylthiazol-2-yl)-2,5-diphenyltetrazolium bromide (MTT), alamarBlue and Calcein AM assays are most commonly used to determine the effect of wound dressing materials on the viability of the tested cells by direct or indirect contact methods [223,224]. In direct contact method wound dressings are placed in direct contact with the tested cells while in indirect contact method extract or leachate of the wound dressings in media is used to treat the cells for specified period of time and then the cell viability assay is performed according to the standard protocols [225,226].In the MTT assay, metabolically active cells reduce the yellow MTT reagent to purple formazan crystals due to the activity of NAD(P)H-dependent oxidoreductase enzymes present in viable cells. Subsequently, the insoluble formazan crystals are dissolved in dimethyl sulfoxide (DMSO) followed by spectrophotometric quantitation at 500–600 nm. If the solution is darker, it means greater number of viable (metabolically active) cell are present. Moreover, cell viability is directly proportional to absorbance values and directly indicates percentage of living cells. Cell viability is generally calculated as a percentage (should be >80%) and compared with control (untreated cells) to ensure the non-cytotoxic nature of dressings materials. The detailed mechanism of alamarBlue assay is discussed under Section 5.4. Cell viability can also be measured using live-dead assay wherein cells treated with dressing material are stained with Calcein AM, and ethidium homodimer-1 that produce green and red colored fluorescence in live and dead cells, respectively. These cells are then visualized under fluorescence/confocal microscope to detect and quantify the presence of the live and deal cells [226,227]. In addition to being non-toxic/cytocompatible the dressing materials should not affect the normal morphology of the cells. Therefore, cell morphology evaluation is carried out along with the cell viability assay. For this purpose, the morphology of the cells treated with dressing is studied using microscopic techniques such inverted light/fluorescence and confocal microscopy) [198]. The live-dead assay kit (Calcein AM dye) can also be utilized to observe morphology of the cells using the fluorescence microscopy [198]. In addition, many other dyes (such asFluorescein diacetate, and DAPI) has also been used by the researcher to study the effect of dressings materials onto the morphologies of the treated cells [228,229].

### 5.3. Cell Attachment/Adhesion

Wound dressings should allow better cell attachment to improve the wound healing process through enhancement of soluble factors secretion and vascularization after cell spreading, cell proliferation, and cell migration. Cell-materials interactions, and subsequent cell adhesion on wound dressings are influenced by the type of polymers utilized, cell binding motifs, mechanical properties, and functionalization of wound dressings materials [216,230]. Better cell attachment on wound dressings ensures the cytocompatibility of the dressing’s materials. Wound dressings are fabricated in different forms such as films, scaffolds, hydrogels, nanofibers, and 3D printed matrices using either natural or synthetic polymeric biomaterials. Natural polymeric based biomaterials are extensively utilized in recent years for fabricating wound dressings due to its biomimetic properties [231,232]. However, synthetic polymeric biomaterials functionalized with cell binding motifs to promote cell adhesion on wound dressings surface are also being utilized for wound healing applications [233]. More recently, plasma treatment of wound dressings surface modulates the surface functionality, hydrophilicity, and wettability to improve the cell attachment on wound dressings [234,235]. The evaluation of cell attachment on wound dressings surface is mainly performed through scanning electron microscopy) [236,237]. In order to investigate the attachment/adhesion of the cells on the surface of the dressing, the dressing material is first sterilized (by exposing to UV light) and then placed in the culture plate containing culture media. The cells are then seeded onto the dressing material and incubated for different time intervals (generally 1 to 7 days). Thereafter, the dressing material is washed, cells are fixed by using glutaraldehyde solution and then dressing dehydrated using different concentrations of alcohol. Finally, the SEM analysis of the dressing materials is performed to study cell attachment and the morphology of the attached cells [236,238,239].

### 5.4. Cell Migration and Proliferation in Wound Dressing Material

Cell migration and proliferation are of paramount importance in the wound healing process as these cellular processes promote the secretion of soluble factors (growth factors, cytokines, inflammatory mediators, and other cellular mediators), vascularization, ECM synthesis, and re-epithelialization [240]. Wound dressings materials’ capability to promote better cell migration and proliferation leads to faster wound closure and healing. Cell migration within wound dressings is evaluated by various microscopic techniques (confocal, fluorescence, etc.). Furthermore, cell proliferation on wound dressings and subsequent secretion of soluble molecules overtime is estimated by various in vitro assays, viz., AlamarBlue assay and enzyme-linked immunosorbent assay (ELISA), respectively [241,242]. AlamarBlue assay quantitatively measures cell viability and cell proliferation using weak fluorescence blue based indicator dye resazurin which works on oxidation-reduction (REDOX) properties of metabolic cells. The blue color of alamarBlue resazurin changes to pink resorufin (strong fluorescent dye) after reduction by viable metabolically active cells followed by measuring fluorescence/absorbance at 570/600 nm. Alamar blue assay can also be utilized for cytokine bioassay. As compared to other reagents used for cell viability and cell proliferation assays, AlamarBlue reagent is advantageous as it is non-radioactive, non-toxic, easy to use, less expensive, provides a fast estimation of cell proliferation of a large number of samples, and can be used to determine cell growth kinetics [243]. Furthermore, ELISA is the most common technique to estimate the various cellular mediators and cytokines secreted by cells during the wound healing process. ELISA assay employs antigen–antibody interactions as a basis to identify and measure important components of wound repair, viz., growth factors, cytokines, and cellular mediators from cell culture supernatants. The most commonly quantified molecules include anti-inflammatory and pro-inflammatory cytokines. Examples of anti-inflammatory cytokines include fibroblast growth factor (FGF), epidermal growth factor (EGF), platelet-derived growth factor (PDGF), vascular endothelial growth factor (VEGF), interleukin-10 (IL-10) and transforming growth factor-beta (TGF-β1), while pro-inflammatory cytokines include interleukin-6 (IL-6), tumor necrosis factor (TNF-α), etc. [244,245].

### 5.5. In Vitro Assay Methods for Evaluation of Wound Healing

During the wound healing process, cells from wound edges start proliferating and migrating into the core of the wounded area leading to re-epithelialization of the wounded surface for restoration of the skin’s barrier function. Moreover, the migration and proliferation phases of the wound healing process represent a limiting event for quality wound healing. Therefore, in vitro wound healing assays are performed to mimic the late inflammatory and proliferative phases of wound healing based on cell migration assays [246]. The wound healing assays are convenient and cost-effective methods for investigation of collective cell migration under different culture conditions. In recent years, various models (2D and 3D) of wound healing have been developed. The advantages of the wound healing assay include qualitative and quantitative estimation of collective cell migration, providing information about molecular mechanisms of the healing process, the effect of cell–matrix and cell–cell interactions, as well as potential therapeutic interventions for improved wound healing. Wound healing assays are generally performed using a two-dimensional (2D) cell monolayer format, wherein the confluent cell monolayer is wounded under highly controlled in vitro conditions followed by the analysis of collective cell migration to ensure healing [247,248,249]. This 2D cell monolayer format is the most widely utilized ethnic alternative to animal models due to its simplicity, faster action, and cost-effectiveness. However, the complexity of the wound healing process is not completely recapitulated by the 2D format, therefore, development of three-dimensional (3D) in vitro skin models are gaining much attention in recent years as a progressive next step for wound healing research. In order to capture the complex wound healing mechanics, 3D skin models or bioengineered skin models employ scaffolds seeded with skin cells to mimic the cell–matrix and cell–cell interactions occurring during wound healing process.

#### 5.5.1. 2D Wound Healing Assays

The basic principle of 2D wound healing assays involves the creation of cell-free region (wounds) in confluent cell monolayer deliberatively followed by monitoring of the wound healing process through analysis of collective cell migration and data acquisition (time-lapse microscopy, impedance measurement) as well as data evaluation [250].

##### Scratch Assay

The most common and well-established 2D in vitro wound healing assay is the scratch assay to assess the cellular and molecular mechanisms of cell migration for wound healing. In this simple and cost-effective method, wound or scratch is generally created mechanically to a cell monolayer followed by quantification of cell migration rate [246,251]. Scratch assay is one of the first developed 2D wound healing assays. In general, scratch is made in cell layer using cell scrapers, pipette tips, toothpicks, metallic micro-intenders, cell culture inserts, or ultraviolet rays [252,253,254]. Thereafter, the cell migration is quantified by measuring the width area, wound width, and relative wound density at defined time intervals using either area method, or wound closure rate method [255]. The advantages of scratch assay include in vivo cell migration mimicking ability, convenient capture of live cell migration, and monitoring of intracellular cellular events [256]. Despite its simplicity and cost-effectiveness, scratch assay suffers from several limitations including requirement of longer time (cell monolayer preparation and scratching), non-uniform cell monolayer, scratch width variation (irregular scratches), mechanical injury to cells and ECM, high resources requirement (cells, test compounds), accumulation of cells near edge of manually created gap, open and static cell culture system, and non-feasibility for high throughput screening [251,257,258]. Cell monolayer mechanical destruction is also possible by skin “stamping” methods. Furthermore, other methods to create wounds for healing assay include electrical, vacuum, PDMS barrier, thermal, laser and optical wounding. In recent years, newer scratching techniques for wound creation have been developed with research progress in this domain. The newer scratching techniques improve the reproducibility of in vitro wound assay as these are useful for creation of uniform scratches (shape and size). Furthermore, the creation of multiple scratches of uniform shape and size in one attempt is possible using these newer techniques. Some of the representative commercially available tools for scratching include AutoScratch™ wound making tool (BioTek), IncuCyte^®^ WoundMaker (Sartorius), HTSScratcher (Peira Scientific Instruments), Cell Comb™ Scratch Assay (Merck), and Wounding Pin Tools (V&P Scientific, Inc.).

##### Skin Stamping-Based Wounding

Wound stamping is another method wherein a stamp mold is placed on top of the cell layer followed by force/pressure application either manually or through an automated process to create wounds in a confluent cell monolayer [259]. In this assay method, a weighted stamp mold is placed on top of cells along with pressure application for the destruction of cells. The engraving through mold can be performed with regular patterns such as parallel lines, squares, or concentric circles. The materials utilized for making mold can be either rubber or polymers such as poly(dimethyl)siloxane (PDMS) [260,261]. The extent of damage depends on the type of mold materials employed for creating wounds. The cells covered by mold are destroyed followed by the removal of remaining cell debris near wound area. Thereafter, the cell migration rate is monitored to evaluate the wound healing process. If cell debris is not removed properly, cell migration can be monitored accordingly. The most commonly utilized mold materials are PDMS as it easily attracts cell debris. This method can also be combined with thermal methods for wound creation (thermo-mechanical method). The advantages of the stamping method include the ability to create different wounds of any shape and size, ability to monitor the influence of cell debris on cell migration, and no damage to proteins/physiological mediators during the wounding process. In addition, cell culture matrix coatings are not affected during the wounding process, unlike scratch assay. The disadvantage with this method includes irregularity in manual pressure to molds like other mechanical wounding methods.

##### Electrical Wounding

Electric or electric cell-substrate impedance sensing (ECIS™) based wounding method employs the combination of biological cell parameters (attachment and spreading) and impedance spectroscopy for wound creation [262]. In order to perform this assay, an electrode is placed at the bottom of a multi-well array containing a confluent cell monolayer [263,264]. Furthermore, an elevated current pulse is applied in the area of the electrode (gold film electrodes), leading to electroporation and eventual cell death for the wound creation [264]. Herein, a constant alternating current is applied on the electrode for monitoring the initial cell growth, wounding, and regrowth of a confluent cell layer. Cell migration rate is determined by measuring electrical impedance and higher electrical impedance represents higher cell migration. The increase in impedance is mediated by the insulating properties of cell membrane for measurement. The advantages of this assay method include real-time cell migration measurement, elimination of human errors via automation, and high reproducibility [262,263]. However, difficulty in detachment of strongly attached cells (fibroblasts, keratinocytes), alteration in cell-cell adhesion and cell-matrix adhesion, high density of cell layer, and sensitivity of impedance with fluctuation of media composition, pH, and temperature represents disadvantages of this method. In addition, specialized equipment ECIS™ is required for this assay, unlike the traditional scratch assay.

##### Microfluidics-Based Assay

**Figure 5:**
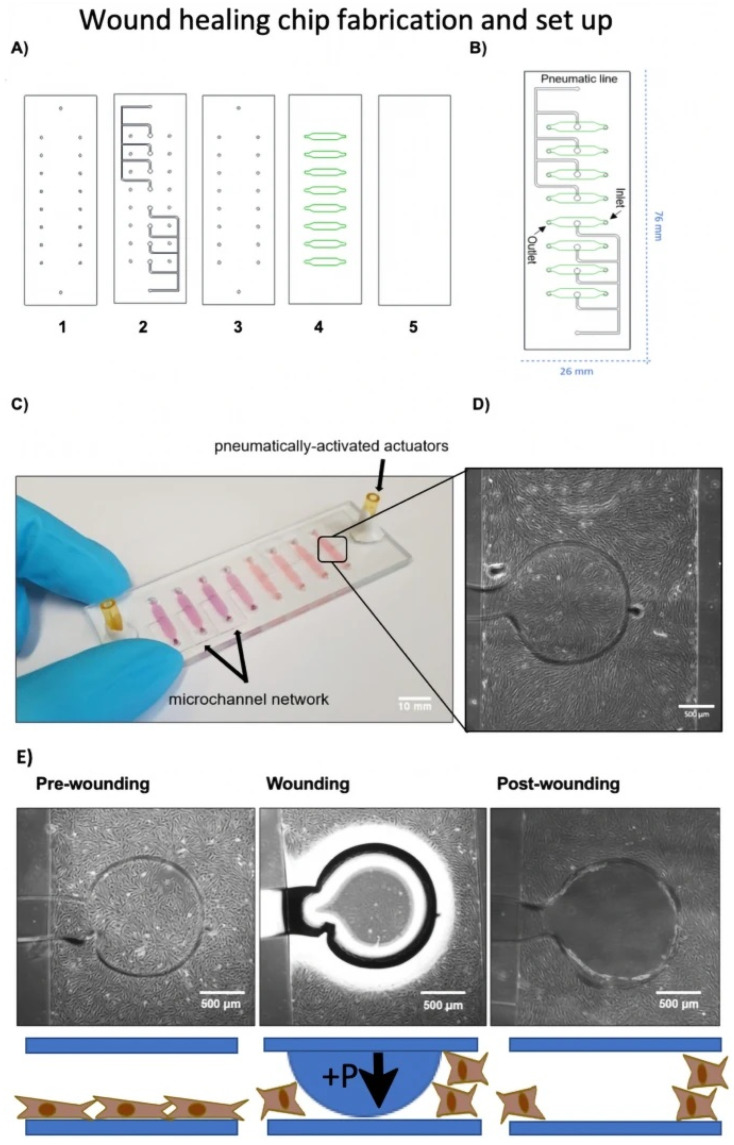
Schematic of microfluidic devices based in vitro wound healing assay. (**A**) Structure of 5 different PDMS layers for fabrication of chip for wound healing assay, (**B**) 2D structure of microfluidic device showing chambers with inlets and outlets, (**C**) Microdevice image showing eight channels filled with pink dye, (**D**) magnified single channel showing defined circular wound actuators located at the center, (**E**) on chip depletion procedure showing the cell monolayer, wounding state via pressure application and post wounding stage indicating cell migration. Reproduced from Ref. [265] with permission from Nature Publishing Group. This work is licensed under a Creative Commons Attribution 4.0 (CC BY) International License.

**Figure 6:**
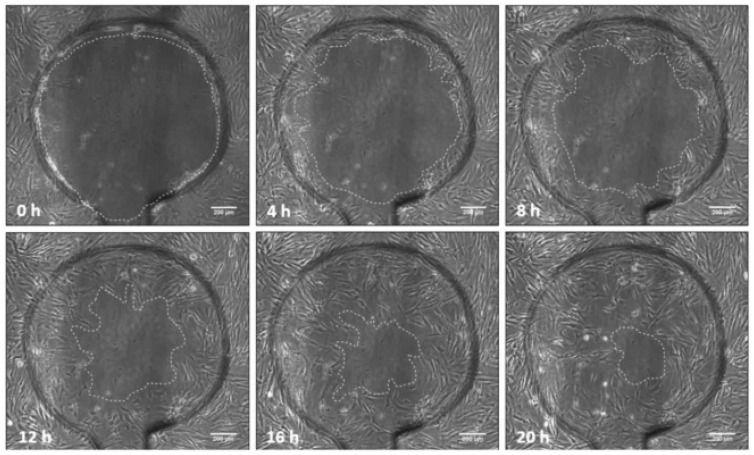
Evaluation of fibroblasts migration and wound closure using microfluidic device based wound assay at different time points under through time lapse images under standard experimental conditions. Reproduced from Ref. [265] with permission from Nature Publishing Group. This work is licensed under a Creative Commons Attribution 4.0 (CC BY) International License.

Microfluidics based wound healing assay relies on cell migration towards wounds using microfluidic devices [265,266]. Microfluidic devices allow cell culture and application of external stimuli in a precise and controlled manner to mimic an in vivo-like microenvironment [258,267]. These devices require a small volume of cells and generally comprise two channels (with inlets and outlets) to provide a miniaturized platform. The main channel contains the culture medium, while the other channel carries wound creating agent (such astrypsin) for cellular detachment in certain area without mixing with each other due to laminar airflow [268]. In this method, wounds are created by enzymatic depletion, depletion, or cell exclusion [266,269]. The trypsin enzyme is most commonly used for creating a scratch followed by its replacement with culture media to allow cell migration into the scratch. The schematic diagram shows a scratch creation and wound healing assessment through the evaluation of cell migration and wound closure using a microfluidic device (Figure 5 and Figure 6).

Apart from analyzing cell migration rate, microfluidics devices’ amenability allows image capturing for monitoring of cells and fluid [267]. The main advantages of microfluidic-based wound assay include the requirement of small volume of cells and culture media, unform monolayer formation, no direct contact with cells or media, precise control of experimental conditions, fully integrated protocol, high reproducibility, no cell substrate, damage, ability to assess the influence of chemicals and mechanical stimulation on wound healing [258,270]. The possible drawbacks of microfluidic devices-based wound assay are requirement of technical expertise, daily media replacement required due to small volume, cell clumps and air bubbles formation, maintenance of controlled humidity, and leakage of the solution from the device.

##### Thermal Wounding

Thermal wounding is achieved by the application of excessive heat to a specific area of a cell monolayer [271]. In this assay method, thermal-mechanical damage is carried out to monitor wounding and regrowth of the cell monolayer. The advantages of this method include the ability to analyze thermo-mechanical damage. However, the wounding area is not limited to defined area as a result of heat application presents a major challenge using this method.

##### Optical (Laser) Wounding

Optical or laser-based wounding represents another wounding method based on the utilization of either infrared (IR) or ultraviolet (UV) lasers for the creation of customized wounds of any shape or size [272,273]. In this assay method, a laser wavelength of 100–315 nm is generally utilized to create a unique wound environment. IR laser-based wounding simulates the thermal damage by producing localized heating in a cell monolayer to mimic skin burns. Stiletto^®^ (Hamilton Thorne) represents one of the commercial IR laser systems for wound creation. The advantages of this method include high reproducibility, high throughput, and a sterile environment. The denaturation of ECM and the production of cell debris due to thermal damage its disadvantages. In addition, the acquisition of a specialized instrument-laser-enabled analysis and processing (LEAP^TM^) is required for performing this assay, unlike scratch assay.

##### Vacuum-Based Wounding

Vacuum based wounding method creates circular wounds by removing specified cell areas with the application of vacuum suction [274]. In this method, small circular wounds are useful for obtaining reproducible measurements as it allows easy relocation of the sample point. In addition, vacuum suction causes less interference with measurements by removing the cell debris from the wounded area. The disadvantages of this assay method include lack of automation and non-uniformity of wound size and shape.

##### Polydimethylsiloxane (PDMS) Barrier-Based Wounding

In this method, a fix-sized barrier commonly made up of PDMS is placed on the culture surface followed by cell growth to form a monolayer. Once a monolayer is formed PDMS based barrier is removed to create a scratch or wound [275]. Herein, a barrier of different sizes and shapes can be utilized to create different types of wounds. The advantages of this assay include a suitable platform to study cell-matrix interactions, standardized wound creation ability of different shape and size, and impenetrable towards physiological mediators and proteins. However, the hydrophilicity of the PDMS barrier does not allow its auto-adherence to the culture surface and may lead to leakage of proteins and cells before removal [276,277]. In addition, automation is difficult with this method as it requires constant attachment and detachment of barrier to the cell surface.

##### Microjets-Based Wounding

Microjets-based wounding is a contactless wound creation method using stationary jets of media. In this assay method, micro-jets of either media or an immiscible fluorocarbon (FC40) are applied onto a monolayer of cells to create any type of 2D pattern wounds in seconds [278]. This method can be automated and multiplexed by integration with microfluidics devices [279,280]. An array of chambers containing wounds in a monolayer can be constructed in very short span of time for wound healing and drug screening. The advantages of this method include ability to create wounds of any shape or size, automation, and high reproducibility.

#### 5.5.2. 3D Wound Healing Assays

Wound healing under in vivo conditions allows cell migration in all directions while surrounded by ECM and other cells, which was not recapitulated by 2D wound healing assays. In order to overcome these limitations, 3D cell culture and wound healing assays are required. The cells cultured in 3D environment completely mimics the in vivo microenvironment in terms of cell morphology, cell migration behavior, signaling and metabolic functions compared to 2D-cultured counterparts [281,282]. Therefore, many efforts are put forward to transfer 2D wound healing assay to 3D wound healing assay to serve the complexity issues of the wound environment. The constructs which are utilized for 3D wound healing assays include in vitro 3D skin models also referred to as bioengineered skin, skin equivalents or artificial skin. Bioengineered skin constructs being utilized for 3D wound healing assay comprise bilayered structure combining both epidermal and dermal components. The dermal counterpart is made from scaffolds embedded with fibroblasts to mimic dermal layer of skin. The scaffolds utilized could be either natural, synthetic polymeric biomaterials, glycosaminoglycans or decellularized dermis. Over the dermal structure, keratinocytes are cultured and matured on the air-liquid interface to form an epidermal counterpart of skin. However, 3D bioengineered skin constructs lack vascular and immune systems as well as appendages (sweat glands and hair follicles). To overcome this limitation, co-cultured and 3D bioprinted skin constructs have been developed to mimic native skin structure along with appendages, and vascular and immune systems using additional types of skin cells [283,284]. Currently, several 3D skin grafts are commercially available for 3D wound healing assays such as Autograft System, Apligraf, Hyalograft 3D, and TissueTech [285]. In order to standardize the 3D wound healing assay procedure, wounds are created using mechanical injuries such as scalpels, punch biopsies, mashers, thermal and electrical instruments after the maturation of 3D construct. The created wounds lie in the range of epidermal injury to full-thickness wounds. However, all these methods suffer from limitations, viz., poor reproducibility, unintentional epidermal layer detachment, etc. To overcome these problems, automated wounding using rotating drills or laser-based devices has been attempted [286,287]. Thereafter, wound healing is qualitatively monitored by immunoassays, histological analysis, and reverse-transcription polymerase chain reaction (RT-PCR) [288]. In addition, confocal microscopy and advanced time-lapse microscopic techniques are utilized for tracking cells in 3D environment [289]. Furthermore, the quantitative wound coverage monitoring is carried out as “percent healing” using NIH ImageJ software by calculating in-depth migration rate [290]. However, the calculation of migration rate in 3D wound healing assays needs further development of standardized and automated analysis for high throughput screenings.

## 6. In Vivo Methods for Evaluation of Wound Dressing Materials

In vivo models or animal models provide invaluable information about wound healing by investigating its cellular and biochemical mechanisms. In addition, these models are the most predictive models for the evaluation of the efficacy and safety of various therapeutic drugs/agents and serve as proper alternative options for wound healing evaluation. The assessment through in vivo models includes the creation of wounds in laboratory animals followed by observation of wound closure and healing over time. A wide range of animal models, viz., rats, mice, rabbits, and pigs have been utilized for in vivo wound healing evaluation using wound dressings. However, due to differences in anatomical and physiological functions of animals and humans, there is no consensus on using a single animal model. The principles of the 3Rs (replacement, reduction, and refinement) should be followed by animal models to ensure the ethical and humane treatment of the animals. The wound healing efficiency is generally affected by the choice of type of wound dressings used, animal models, location of wound, and microbiome [291]. Once a suitable animal model is determined, the selection of appropriate and reproducible methods needs to be conducted in order to monitor the wound healing progress over time. The in vivo wound healing rate assessment methods employ non-invasive protocols such as wound tracing, biophysical assessment, biochemical assays, histological/immunohistochemical studies, image analysis, and documentation using wound biopsies [292]. Currently, there is a wide range of animal models and methods that are available for wound healing assessment using wound dressings. In this section, an overview of most frequently utilized in vivo models and methods for wound healing evaluation is reviewed.

### 6.1. Partial Thickness Wound Models

A partial thickness wound is defined as damage to the epidermis and sometimes superficial dermis but no damage to the basement membrane and dermal blood vessels. In order to create a partial thickness wound model, various methods such as tape striping, blister, and abrasion techniques are utilized. Tape striping is the simplest partial thickness technique to remove only the superficial skin layer (stratum corneum) using adhesive tape [293]. The wounds created by this method are affected by the adhesiveness of the tape and exerted manual pressure. The advantage of this model includes simplicity, relative painlessness, and less harm to the skin. This model is utilized for the evaluation of re-epithelialization, epidermal growth kinetics, and the adhesive nature of wound dressings in dermo-pathological research. The disadvantage of this model is the difficulty to maintain consistency of the wound as this model is limited only to the superficial layer. Another technique to create partial thickness wounds includes blistering which is formed by separating epidermis and dermis layer. Blisters are induced by using various mechanical suction devices, heat, and chemical, or biological vesicants [294,295,296,297]. The blister-based partial thickness wound models are utilized for investigating wound healing kinetics, cell migration/proliferation, and long-term epidermal regeneration [294,298,299]. Wound healing using this model is assessed by measuring various parameters such as trans-epidermal water loss wound area by quantitative image analysis. In a recent clinical study, bacterial nanocellulose-based wound dressings (epicite^hydro^) have been utilized for wound healing evaluation in partial thickness burns and indicated wound healing with less exudate, decreased pain, and scarless healing [300]. Furthermore, the abrasive wound model is created by inflicting uniform superficial abrasions using surgical brushes for wound healing evaluation of a variety of wound care products (for example, wound dressings). In this model, only the superficial epidermal layer is removed with an intact basement membrane and is comparable to the suction blister model in terms of wound depth. As the basement membrane is intact in this model, it promotes wound healing without scarring. In a previous study, topical antimicrobial gel-based dressings were evaluated for wound healing evaluation [301]. This model has been utilized for wound healing evaluation using wound dressings (polyurethane, hydrogel, hydrocolloid) in various randomized clinical studies [302]. The results of clinical studies indicated enhanced healing ability of wound dressings that provide a moist wound healing environment.

### 6.2. Full Thickness Wound Models

Full thickness wounds extend beyond the epidermal and dermal layer to the subcutaneous layer and involve complete removal of the epidermis and dermis and disrupt the basement membrane and dermal blood vessels. The full thickness wounds are created by using different devices, viz., scalpel, dermatome, punch biopsy, and laser with precise control over size and depth of wounds. This model is utilized for the healing of both epidermal and all dermal components by investigating angiogenesis, wound contraction, and wound closure [303,304]. In addition, the effect of wound dressings and new therapeutic treatments on wound healing can also be investigated. The methods employed for assessment of wound healing rate include measurement of total excisional volume, biochemical assays (collagen or proteoglycans), angiogenesis assessment, histological studies to analyze the granulation tissue formation, and re-epithelialization. In a recent study, Kuo et al., evaluated the wound healing ability of film-based dressings in partial and full thickness pig wound model [305]. The results indicated complete re-epithelialization, blood flow signals, and wound healing as revealed by wound closure and histological studies.

### 6.3. Surgical Wound Model

Surgical wounds are wounds that are made through a cut using a scalpel during surgery. These wound models are classified into excisional and incisional wound models.

#### 6.3.1. Excision Wound Models

Excision wound models are the most commonly utilized wound models and are generated by surgical removal of all skin layers (epidermis, dermis, and hypodermis) from animals. This model is a full thickness injury wherein skin edges are not sutured together to resemble clinical wounds [306]. In this model, an impression is made (not more than 2 mm depth) on the dorsal thoracic region of animals after anesthesia to remove a 300 mm^2^ circular area using surgical blades and scissors [307]. This model is useful in the investigation of re-epithelialization, granular tissue formation, angiogenesis, and ECM remodeling during wound healing using different methods. The evaluation parameters for the excisional wound model include wound area change measurement, epithelialization period determination, wound index, collagen estimation, and protein estimation. The methods utilized for wound healing assessment include macroscopic observation of change in the wound area using a camera, calculation of wound closure (wound healing rate), wound contraction area measurement, wound index, biochemical assays (collagen and glycosaminoglycans estimation), histological studies (ECM synthesis) [308,309,310]. In a recent study, Mukherjee et al., utilized chitosan-based hydrogel based wound dressings for the evaluation of wound healing in the excisional wound rat model [311]. The results indicated enhanced wound healing in the presence of Pluronic F68 within 15 days.

#### 6.3.2. Incisional Wound Model

The incisional wound model represents the second most common wound model for evaluation of wound healing. This model is beneficial for the investigation of surgical incision materials (sutures, dressings) such as mechanical properties and degradation properties for effective wound healing potential [312]. In this model, a longitudinal incision/multiple incision is made parallel to the midline of the wound on the dorsal side of the animals passing through all skin layers (epidermis, dermis, and subcutaneous tissue) to the muscle [313]. Incisional wounds can be further classified into two types, viz., primary (first intention) or secondary closure (second intention) depending upon the action. In the case of the primary closure model, wound infliction is immediately sutured, followed by the application of dressings. The primary closure model serves as an excellent model for the analysis of biomechanical properties and is less suitable for histological assessment of wound healing. On the other hand, skin incisions are not closed immediately in the case of the secondary closure model and are utilized for evaluation of wound healing activities through histological assessment, biochemical assays, epithelialization period (re-epithelialization), and scarring [314]. In an earlier clinical study, three different semi-occlusive film-based wound dressings were utilized as a protective cover for 3637 surgical incisions cases for eight years and demonstrated faster wound healing as indicated by visual wound assessment, decreased pain, and less scarring [315]. Furthermore, alginate and other polymeric hydrogels-based wound dressings have also shown effectiveness in managing surgical incisions [316,317]. In another study, chlorhexidine gluconate (CHG) film-based antimicrobial wound dressings have shown effectiveness with strong antimicrobial activity and wound healing properties in post-operative wounds in the porcine incisional wound model [318].

### 6.4. Burn Wound Model

Burn wound healing models are generated in animals using either thermal damage or water scalding of the skin. In the thermal damage model, direct application of heat is conducted through a hot metal plate, or cylindrical metal rod, while in water scalding model, blisters are created in the skin by exposing it to hot water [306]. In both these models, the dermis is exposed, leaving an open wound of either partial thickness or full thickness [308]. Furthermore, the extent of burned area depends on treatment methods applied and its time/duration. Wound dressings are applied after burn wound generation to evaluate the wound healing properties. The burn wounds model is utilized for the analysis of wound contraction, re-epithelialization, vascularization, wound tissue biochemistry, granulation tissue formation, and scarring during wound healing depending on the depth of the burn. Based on the depth of the burn, burn wounds are categorized into first degree (superficial-thickness), second degree (partial-thickness), and third degree (full-thickness) as demonstrated by [319]. The methods utilized for the assessment of wound healing properties include visual assessment via digital photographs, histopathological and immunohistological analyses for estimation of ECM (collagen fibers) deposition, vascularization, and granulation tissue formation. The analysis of wound healing over time is performed by myeloperoxidase assay (MPO). She et al., utilized collagen foam-based wound dressings in a rabbit burn wound model and demonstrated effective and scarless burn wound healing with better wound recovery [320]. In recent years, hydrogels-based wound dressings have been utilized for burn wound management as cooling alternative first aid dressings [321]. The cooling effect provided by hydrogel-based dressings varies with the depth of the burn wound. The temperature provided by hydrogels at a depth of 1–3 mm is about 33 °C, whereas on the surface of wound around 20.5 °C [322]. Hydrogels-based dressings not only cool the burn wound but also reduce pain and contamination in the wound area to prevent further injuries.

### 6.5. Diabetic Wound Model

Diabetic wound models are generated in animals to clinically resemble diabetic ulcers for wound healing evaluation. These wound models are created in animals after inducing diabetes with chemicals (alloxan or streptozotocin), dietary induction (high-fat diet), surgical manipulation, or through genetic means (systemic mutations) [292]. Among all these methods, a chemicals-based method and high-fat diet are most commonly utilized for diabetes induction in preclinical studies. So far, no single diabetic model can recapitulate the complexity of the diabetic pathological process. Currently developed diabetic models mimic merely one or few aspects of this complex multifactorial disease and mostly utilize the hyperglycemic condition of diabetes for induction and further assessment [251]. These models can be utilized for creating multiple wounds per animal and provide a platform to test pharmacological agents. However, variations in different diabetic models and their inability to mimic human diabetic complications possess major challenges. In an earlier study, various wound dressings such as hydrocolloid dressings, foam-based dressings and hydrogels dressings have been compared for wound healing efficacy in various diabetic models and indicated the hydrogels-based dressings as the most efficacious one [323]. In another study, chitosan hydrogel-based wound dressings incorporated with exosomes derived from mesenchymal stem cells demonstrated improved wound healing in the full thickness diabetic model as revealed through enhanced re-epithelialization, collagen deposition, and vascularization [324]. In a recent study, silk fibroin scaffolds based wound dressings demonstrated accelerated wound healing in a diabetic rat model [325].

### 6.6. Infected Wound Model

**Figure 7:**
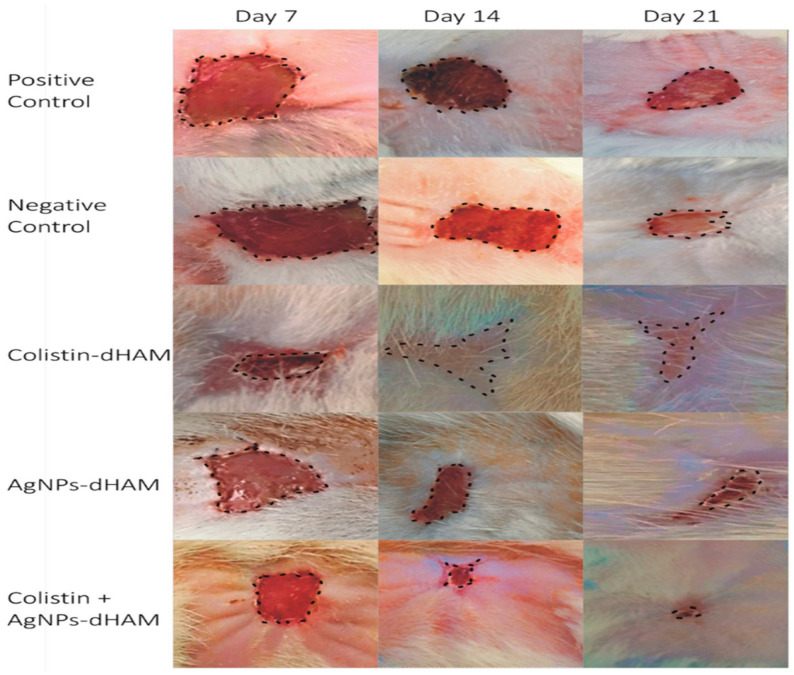
Wound healing evaluation against *Pseudomonas aeruginosa* in burn wound infected rat model at different time points using different wound dressings. The wound dressings employed for healing assessment comprises decellularized human amniotic membrane (dHAM) containing colistin and/or silver nanoparticles (AgNPs). Reproduced from the ref. [328] with permission from Nature Publishing Group. This work is licensed under a Creative Commons Attribution 4.0 (CC BY) International License.

The infected wound models clinically resemble infected diabetic wounds, infected-burns, and other infected ulcers. In this method, specific organisms, or group of organisms with biofilm-forming ability are inoculated to create infected wounds [326]. In order to do so, firstly wounds are created using excision or incision followed by inoculation using specific organisms. Occlusive wound dressings should be utilized for infected wounds to provide optimal conditions for bacterial growth and prevent cross-contamination. This model is utilized for studying host and pathogens relationships as well as complexity of immune responses toward infection during the wound healing. In addition, the antibacterial effects of new drugs can also be studied using this model. However, significant limitations in utilizing this model include difficulty in getting ethical approval as this model requires pathogenic organisms. Antimicrobial wound dressings (wound dressings with antimicrobial properties) have shown tremendous application in the treatment of infected wounds. These dressings are prepared by incorporating antibiotics, chitosan, essential oil, and nanoparticles of honey [25]. Anisha and co-investigators utilized antimicrobial sponge based wound dressings comprising chitosan, hyaluronic acid, and nano-silver for the management of infected diabetic foot ulcers. The results demonstrated strong antibacterial activity of developed antimicrobial dressings against *E. coli*, *S. aureus*, *P. aeruginosa*, and *K. pneumonia*, and methicillin-resistant *Staphylococcus aureus* (MRSA) [327]. In a recent study, silver nanoparticles and colistin-impregnated decellularized human amniotic membrane wound dressings demonstrated synergistic effects as revealed by outstanding antibacterial activity and enhanced wound healing in a burn wound-infected rat model (Figure 7).

### 6.7. Methods Utilized for Wound Healing Assessment Using In Vivo Wound Models

The understanding of factors affecting wound healing process, wound pathophysiology, and suitable conditions required at the wound bed are necessary for appropriate wound assessment and increased therapeutic effectiveness. Therefore, a comprehensive analysis of progressive changes occurring during the wound healing process should be carefully monitored, estimated, and documented. Currently, a wide range of qualitative and quantitative methods are available to evaluate several parameters related to the progression of wound healing using wound dressings through several techniques. The common methods for evaluation of wound healing using in vivo wound models using wound dressings include visual inspection for wound size changes measurement, epithelialization, vascularization and ECM deposition using wound healing rate analysis, biochemical assays (collagen metabolism, oxidative stress, myeloperoxidase), and histological and immunohistochemical analysis (cytokines and growth factors release) [292]. All these measurement methods provide information regarding the wound bed characteristics, tissue growth, extent of scarring, vascularization, and pathological disorders. The wound healing assessment methods are further divided into two types-non-invasive methods and invasive methods based on their procedure. The non-invasive methods include visual macroscopic observation comprising wound analysis by imaging (normal photography, image analysis software), wound healing rate (change in wound surface area, wound tracing method), and biophysical wound assessment using in vivo imaging methods such as optical coherence tomography (OCT), diffuse near-infrared spectroscopy, and confocal laser scanning microscopy, etc. [329]. On the other hand, invasive methods for wound assessment include biochemical, histological, and immunological methods. Among biochemical assays, there are several methods involved for measuring various biomolecules such as hydroxyproline assay (collagen estimation), oxidative stress profiling (reactive oxygen and nitrogen quantitation), myeloperoxidase assay (evaluation of inflammatory phase and neutrophils recruitment/accumulation), and N-acetylglucosaminidase (macrophages assessment) [330,331]. Histological methods are utilized for qualitative assessment of pathological conditions and wound healing progression using the most common hematoxylin and eosin staining [325]. Subsequently, immunological methods include immunohistochemical studies (various staining methods using monoclonal antibodies for collagen localization, and re-epithelialization assessment) and ELISA assay (determination of various inflammatory mediators, growth factors and cytokines) [332]. Other methods such as flow cytometry and macrophage polarization studies are also carried out to understand the cellular functions during wound healing. Overall, all of these invasive and non-invasive methods provide comprehensive information about the progression of wound healing.

## 7. Conclusions and Future Perspective

Wound dressings have been utilized for centuries, and with the development of various modern wound dressings in recent years, a better understanding of the wound healing process is realizable. Any specific or single type of wound dressings cannot be applied in a one-size-fits-all manner due to variations in cellular and metabolic functions in different types of wounds. Keeping this in mind, a variety of modern wound dressings are developed to overcome the limitations associated with traditional wound dressings. In this review, we have discussed the different types of modern wound dressings, viz., hydrogel-based dressings, nanofibers-based dressings, cell-based dressings, antimicrobial dressings, stimuli-responsive smart wound dressings, and others with their advantages and challenges. Thereafter, various in vitro wound healing assays and in vivo wound models are described. Furthermore, various in vitro and in vivo characterization methods have been discussed in detail to understand the various parameters and the overall wound healing progression. Despite significant improvement in wound healing using modern wound dressings, there are a few challenges which need to be addressed to develop wound dressings for enhanced therapeutic efficiency. The challenges associated with modern wound dressings include the development of multifunctional wound dressings without compromising the individual component’s properties, and biosafety of developed wound dressings as multiple biomolecules, viz., nanoparticles, peptides, and growth factors are incorporated in dressings. The other challenges include scalability and commercialization issues as most of the modern wound dressings are newly developed and still have a long way to go to reach clinical settings. In addition, scaling up of wound dressings is posing a challenge as many of the modern wound dressings are still in the experimental stage [73]. Furthermore, future trends and outlook related to modern wound dressings should address the above mentioned challenges. Nevertheless, more exploration of materials and technology would be required in the future to develop improved and effective wound dressings. Future trends should focus on exploring the development of multifunctional smart wound dressings with real-time monitoring ability using state-of-the-art characterization, while healing evaluation methods to determine wound status and progression of wound healing in one go also present an interesting perspective [18]. In addition, the integration of electronics and telemedicine with modern wound dressings for personalized therapeutics can be a future direction.

## Figures and Tables

**Table 1:** A representative list of modern wound dressings for acute and chronic wounds healing.

Wound Dressings	Biomaterials	Type of Wounds	Type of Study	Findings	Ref.
Film based dressings	Castor oil loaded with chitosan modified zinc oxide nanoparticles	Acute	In vitro and in vivo	Enhanced dynamic mechanical performance, strong antimicrobial effects, faster wound healing	[82]
Film based dressings	Polyurethane/poly(N-vinylpyrrolidone) composite	Acute (full thickness)	In vitro and in vivo	Increased water absorption, high water vapour transmission rate, enhanced re-epithelialization	[83]
Film based dressings	Hyaluronic acid	Chronic (perianal wound)	In vivo	Accelerated re-epithelialization, enhanced fibroblastic proliferation and collagen deposition	[84]
Film based dressings	Sodium hyaluronate loaded with glycyrrhetinic acid	Acute	In vitro	Improved hydration ability, induce cell proliferation and cell migration, rapid wound closure	[26]
Film based dressings	β-Glucan paramylon	Acute	In vivo	Suppression of elevated inflammatory cytokines, significantly faster wound closure	[85]
Foams based dressing	Hydrocellular foam containing silver sulfadiazine	Chronic	In vivo (clinical trial)	Reduction in infection and exudates level	[86]
Foams based dressing	Oxidized cellulose/collagen matrix	Chronic (pressure ulcers)	In vivo (clinical trial)	Decreased level of elastase and plasmin in wound exudates, faster wound closure and healing	[87]
Foams based dressing	Curdlan, chitosan, agarose	Chronic	In vitro	Highly porous structure with superabsorbent properties, high water vapor transmission rate 1700–1800 g/m^2^/day	[88]
Foams based dressing	Silicone	Chronic (pressure ulcers)	In vivo (clinical trial)	Improved wound healing	[89]
Foams based dressing	Silver foam	Chronic (diabetic foot ulcer)	In vivo (clinical trial)	Significantly reduced ulcer area, superior wound healing rate	[90]
Hydrocolloid based dressings	Sodium carboxymethyl cellulose, gelatin, pectin, and adhesive polymers (DuoDERM)	Chronic (pediatric burn injury)	In vivo (clinical trial)	Occlusive moist environment, improved wound healing	[91]
Hydrocolloid based dressings	Gum resin myrrh (Miraderm)	Acute (full thickness)	In vitro and in vivo	Good biocompatibility and biodegradability, improved dermal wound healing	[92]
Hydrocolloid based dressings	Styrene-isoprene-styrene copolymer and polyisobutylene	Chronic (diabetic)	In vitro and in vivo	Increased bioadhesive force and mechanical strength,	[93]
Hydrocolloid based dressings	Alginate loaded with ibuprofen	Acute (suppurating wounds)	In vitro and in vivo	Superior mechanical and rheological properties, faster granulation tissue formation, significantly higher healing rate	[94]
Hydrocolloid based dressings	alginate, chitin/chitosan and fucoidan sheet	Chronic (diabetic)	In vitro and in vivo	Advanced granulation tissue and capillary formations, improved wound healing	[95]
Hydrogels based dressings	Acrylate-end capped urethane	Acute	In vitro and in vivo	Significant improvement in wound contraction and wound fraction percentages, accelerated wound healing	[96]
Hydrogels based dressings	Lignin hydrogels incorporated with silver nanoparticles	Chronic	In vitro and in vivo	Strong antibacterial and antioxidant activity, lack of wound inflammation, complete tissue remodeling and skin integrity restoration	[97]
Hydrogels based dressings	Gelatin-methacryloyl incorporated with graphene oxide	Chronic	In vitro and in vivo	High exudate-absorbing capacity, increased cell migration and proliferation, enhanced angiogenesis	[98]
Hydrogels based dressings	Quaternized chitosan and benzaldehyde terminated Pluronic^®^F127	Acute (full thickness)	In vitro and in vivo	Suitable stretchable and compressive property, fast self-healing ability, higher thickness of granulation tissue and enhanced collagen disposition	[99]
Hydrogels based dressings	Collagen peptide-functionalized with carboxymethyl chitosan and methacrylate sodium alginate	Acute (full thickness)	In vitro and in vivo	Decreased inflammatory process, improved vascularization collagen deposition, and	[100]
Nanofibrous dressings	PLGA loaded with aloe vera extract and recombinant human epidermal growth factor	Acute (full thickness)	In vitro and in vivo	Improved fibroblast proliferation, enhanced re-epithelization significant accelerated wound closure	[101]
Nanofibrous dressings	Recombinant spider silk protein loaded with endothelial progenitor cells and sodium hydrogen sulfide	Acute	In vitro and in vivo	High hemocompatibility and cytocompatibility, significantly enhanced wound regeneration efficiency	[102]
Nanofibrous dressings	Polycaprolactone/Keratin/platelet-rich fibrin	Acute	In vitro and in vivo	Strong antimicrobial effect, enhanced vascularization and collagen deposition, formation of skin appendages	[103]
Nanofibrous dressings	Liginin	Acute (full thickness)	In vitro and in vivo	Suitable viscosity, accelerated wound closure, collagen deposition, and angiogenesis, increased re-epithelialization	[104]
Nanofibrous dressings	Polycaprolactone-/polyvinyl alcohol-silk fibroin loaded with curcumin	Acute	In vitro and in vivo	Biodegradability, moisture retaining capability, rapid wound closure and healing	[105]
Cell based dressings	Gelatin containing adipocytes derived stem cells	Acute	In vivo	Higher expression of cytokeratin, enhanced re-epithelialization and collagen deposition	[106]
Cell based dressings	Silk fibroin containing human Wharton’s jelly MSCs	Chronic	In vivo	Reduction in formation of fibrotic scar tissue, generation of vascularized granulation tissue	[107]
Cell based dressings	Polycaprolactone containing bone marrow derived MSCs	Chronic (diabetic)	In vitro and in vivo	Reduced expression of pro-inflammatory cytokines with inhibition of M_1_-type macrophages formation, enhanced granulation tissue formation, vascularization, and collagen deposition	[108]
Cell based dressings	GelMA and methacrylated hyaluronic acid containing adipose derived MSCs	Acute	In vitro and in vivo	Increased vascularization, enhanced dermal wound healing	[109]
Cell based dressings	Gelatin, sericin, laminin containing adipose derived MSCs	Chronic (diabetic ulcer)	In vitro and in vivo	Enhanced cell viability and fibroblasts metabolic index, improved tube formation of endothelial cells	[110]
Antimicrobial dressings	Sodium alginate and hardystonite hydrogel	Chronic	In vitro and in vivo	Stimulated proliferation of human dermal fibroblasts and endothelial cells, enhanced vascularization, and re-epithelialization	[111]
Antimicrobial dressings	Bisphosphonate modified supramolecular hyaluronic acid hydrogels with self-healing property	Acute (full thickness)	In vitro and in vivo	Strong antimicrobial activity, faster wound closure rate and re-epithelialization	[112]
Antimicrobial dressings	Hyaluronic acid hydrogels incorporated with Fe ions	Acute	In vitro and in vivo	Inhibition of microbial infections, promotion of cutaneous regeneration	[113]
Antimicrobial dressings	Polycaprolactone nanofibers loaded with thymol	Acute	In vitro and in vivo	Reduction of bacterial load, promotion of keratinocytes and fibroblasts migration and proliferation	[114]
Antimicrobial dressings	Antimicrobial heptapeptide (IKYLSVN) hydrogel loaded with glucose oxidase	Chronic (diabetic foot ulcers)	In vitro and in vivo	Outstanding antimicrobial activity, reduction of glucose concentration	[115]
Stimuli responsive smart dressing	Acryloyl-6-aminocaproic acid (AA) and AA-g-N-hydroxy succinimide hydrogel (pH-responsive)	Acute	In vitro and in vivo	Good hemostatic performance, enhanced collagen deposition and vascularization	[116]
Stimuli responsive smart dressing	Sodium alginate and bFGF-loaded poly(N-isopropylacrylamide) nanogels (temperature responsive)	Acute	In vitro and in vivo	Significant wound contraction (96%), less inflammation and higher angiogenesis	[117]
Stimuli responsive smart dressing	Polydopamine nanosheets loaded with NO donor (Near-infrared light-responsive)	Acute (full thickness)	In vitro and in vivo	Outstanding antibacterial properties, accelerated wound healing	[118]
Stimuli responsive smart dressing	Mesoporous silica nanoparticles decorated with ceria nanocrystals (ROS responsive-H_2_O_2_ mediated)	Acute	In vitro and in vivo	Accelerated wound healing with marked skin appendages formation, reduced scar formation	[119]
Stimuli responsive smart dressing	Guar gum based conducting slime (Conductivity responsive)	Acute	In vitro and in vivo	Self-healing property, accelerated wound healing	[120]
3D printed wound dressings	Chitosan	Chronic (diabetic)	In vitro and in vivo	Significantly higher cell growth and proliferation, improvement in quality of restored tissue	[121]
3D bioprinted wound dressings	Alginate, PRP and PPP loaded with dermal fibroblasts	Acute	In vitro	Enhanced angiogenic, immunomodulatory properties, and ECM deposition, faster wound closure	[122]
3D bioprinted wound dressings	Collagen, fibrinogen, and thrombin containing fibroblasts and keratinocytes (Mobile in situ skin bioprinting)	Acute	In vitro and in vivo	Enhanced keratinocytes proliferation, rapid wound closure rate, reduced wound contraction, enhanced re-epithelialization, vascularization and collagen deposition	[123]
3D bioprinted wound dressings	Collagen containing fibroblasts, keratinocytes, pericytes and endothelial cells	Acute	In vitro and in vivo	Enhanced vascularization, maturation of keratinocytes to form multilayered barrier	[124]
3D bioprinted wound dressings	Skin-derived extracellular matrix containing fibroblasts and keratinocytes	Acute	In vitro	Improved dermal ECM secretion, epidermal organization, and barrier function	[125]

Abbreviations: NO: Nitric oxide, ROS: Reactive oxygen species, bFGF: b-Fibroblast growth factor, H_2_O_2_: Hydrogen peroxide, ECM: Extracellular matrix, MSCs: Mesenchymal stem cells, GelMA: Gelatin methacryloyl, PRP: Platelet rich plasma, PRP: Platelet poor plasma, 3D: Three dimensional.

**Table 2:** A list of clinical and case studies using cell based wound dressings containing mesenchymal stem cells for chronic wounds.

Type of Wound Dressings	Commercial Name	Type of Wound	Findings	Clinical Trial Status and No. of Patients Included	Ref.
Collagen containing bone marrow derived stem cells	Terudermis^®^	Chronic leg ulcer	Successful wound closure with granulation tissue formation	Case report (n = 1)	[152]
Collagen containing bone marrow derived stem cells	Pelnac™	Chronic venous ulcers, burn	Complete healing of burn patients, 2 patients healed after two applications, 3 healed within 3 weeks, 11 patients completely healed within 8 weeks, 2 died before clinical trial end due to unrelated pathology	Phase I (n = 20)	[153]
Collagen containing bone marrow derived stem cells	Surgicoll	Diabetic foot ulcers	Complete healing in 3 patients and significantly reduced wound closure in 5 patients	Phase I (n = 8)	[154]
Polyurethane containing adipose derived stem cells	-----	Diabetic foot ulcers	Complete wound closure in 73% at 8 weeks and 82% patients completely healed at 12 weeks	Phase I/II (n = 59)	[155]
Placenta-derived mesenchymal stem cells hydrogel	-----	Diabetic foot ulcers	Complete healing with no recurrence up to 6 months	Case report (n = 1)	[156]
Decellularized amniotic membrane containing Wharton Jelly stem cells and dermal fibroblasts	-----	Chronic ulcers	Significant decrease in wound size, total skin regeneration with re-epithelialization, progressive wound healing rate (93.92%)	Case study (n = 5)	[157]

## Data Availability

All data generated and analyzed during this study are included in this article.
